# N-WASP Control of LPAR1 Trafficking Establishes Response to Self-Generated LPA Gradients to Promote Pancreatic Cancer Cell Metastasis

**DOI:** 10.1016/j.devcel.2019.09.018

**Published:** 2019-11-18

**Authors:** Amelie Juin, Heather J. Spence, Kirsty J. Martin, Ewan McGhee, Matthew Neilson, Marie F.A. Cutiongco, Nikolaj Gadegaard, Gillian Mackay, Loic Fort, Sergio Lilla, Gabriela Kalna, Peter Thomason, Yvette W.H. Koh, Jim C. Norman, Robert H. Insall, Laura M. Machesky

**Affiliations:** 1 CRUK Beatson Institute, Glasgow G61 1BD, UK; 2 Division of Biomedical Engineering, School of Engineering, University of Glasgow, Glasgow G12 8LT, UK; 3 Institute of Cancer Sciences, University of Glasgow, Glasgow G61 1BD, UK

**Keywords:** pancreatic cancer metastasis, cancer invasion, chemotaxis, cancer cell signaling, cell migration, receptor recycling, endocytosis, actin dynamics, tumor invasion, self-generated gradients

## Abstract

Pancreatic ductal adenocarcinoma is one of the most invasive and metastatic cancers and has a dismal 5-year survival rate. We show that N-WASP drives pancreatic cancer metastasis, with roles in both chemotaxis and matrix remodeling. lysophosphatidic acid, a signaling lipid abundant in blood and ascites fluid, is both a mitogen and chemoattractant for cancer cells. Pancreatic cancer cells break lysophosphatidic acid down as they respond to it, setting up a self-generated gradient driving tumor egress. N-WASP-depleted cells do not recognize lysophosphatidic acid gradients, leading to altered RhoA activation, decreased contractility and traction forces, and reduced metastasis. We describe a signaling loop whereby N-WASP and the endocytic adapter SNX18 promote lysophosphatidic acid-induced RhoA-mediated contractility and force generation by controlling lysophosphatidic acid receptor recycling and preventing degradation. This chemotactic loop drives collagen remodeling, tumor invasion, and metastasis and could be an important target against pancreatic cancer spread.

## Highlights

• N-WASP is a crucial mediator of pancreatic ductal adenocarcinoma metastasis
• Pancreatic cancer cells respond to self-generated gradients of LPA, driving tumor egress
• N-WASP controls trafficking of LPAR1 receptor toward recycling versus degradation
• N-WASP and LPAR1 trafficking control contractility, matrix remodeling, and invasion

## Graphical Abstract

**Figure:**
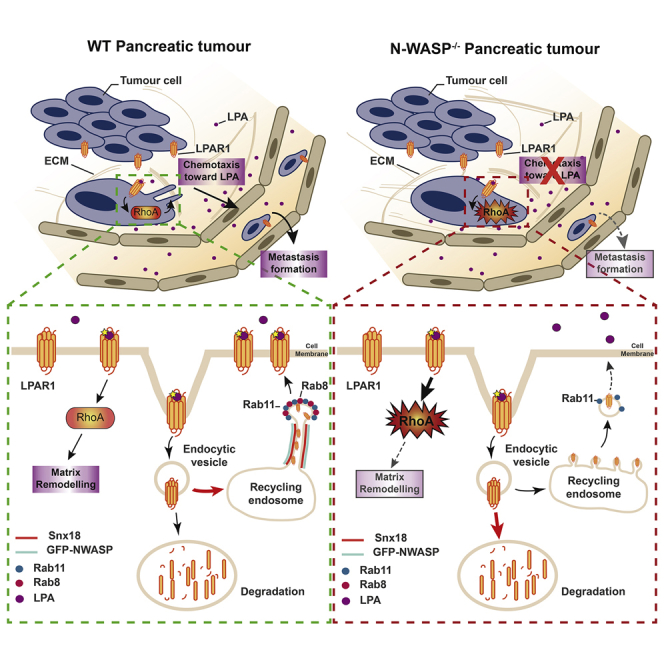

## Teaser

Pancreatic ductal adenocarcinoma is highly metastatic; recycling of the receptor LPAR1 drives metastasis in response to self-generated chemotactic gradients of LPA. Juin et al. show that the signaling adapter protein N-WASP coordinates recycling of LPAR1 back to the cell surface after internalization, driving efficient matrix remodeling, invasion, and tumor egress.

## Introduction

Pancreatic ductal adenocarcinoma (PDAC) is the most common pancreatic malignancy, which unfortunately shows poor response to existing chemotherapies and high incidence of recurrence and metastasis. In most cases, the tumor is not surgically resectable, leading to a 5-year survival of only 5% (He et al., 2014). PDAC tumors show extensive fibrotic stroma containing immune cells, fibroblasts, cancer cells, and matrix (Suklabaidya et al., 2018, Vennin et al., 2018). PDAC extracellular matrix (ECM) components include type I collagen, hyaluronan, and laminin (Liang et al., 2017). Stiffening ECM occurs due to remodeling and activates a cascade of signaling pathways, including focal adhesion kinase (FAK) and JAK-STAT3-Rho kinase, promoting tumor progression and invasion (Rath et al., 2017, Jiang et al., 2016, Laklai et al., 2016). Inhibition of cell contractility using inhibitors of JAK or FAK, ruxolitinib, and VS-4718, respectively, or Rho-kinase (with AT13148) significantly reduces ECM deposition and tumor invasion in PDAC mouse models (Jiang et al., 2016, Laklai et al., 2016). Thus, understanding how cell migration pathways drive tumor cell invasion and metastasis is of major clinical relevance.

Invasion of local tissues by malignant cells relies on cell migration and ECM remodeling and degradation. N-WASP (Neural Wiskott-Aldrich Syndrome Protein) is a ubiquitously expressed actin nucleation promoting protein. N-WASP promotes branched actin assembly via the Arp2/3 complex, leading to membrane protrusion coupled with matrix degradation and invasion of cancer cells into ECM. It supports invadopodia formation (Yamaguchi et al., 2005) and promotes cancer cell invasion (Gligorijevic et al., 2012, Yu et al., 2012). Additionally, N-WASP is implicated in clathrin-mediated endocytosis, organizing branched actin at nascent clathrin-coated pits and aiding internalization (Benesch et al., 2005).

High levels of N-WASP expression correlate with progression and/or poor outcome in human lung cancer (Frugtniet et al., 2017), PDAC (Guo et al., 2014), hepatocellular carcinoma (Jin et al., 2013), invasive breast ductal carcinoma (Yu et al., 2012), and esophageal squamous cell carcinoma (Wang et al., 2010). N-WASP was implicated in invasion and metastasis in a murine breast cancer model (Gligorijevic et al., 2012), but surprisingly, deletion of N-WASP in a murine model of colorectal cancer accelerated tumorigenesis (Morris et al., 2018). The role of N-WASP in tumor progression and dissemination merit further investigation, as it is a promising target against metastasis.

Chemotactic migration through the microenvironment surrounding a tumor allows escape and dissemination (Friedl and Alexander, 2011, Roussos et al., 2011). Recently, lysophosphatidic acid (LPA) was identified as a driver of melanoma cell chemotaxis and invasion (Muinonen-Martin et al., 2014). LPA is rapidly consumed by melanoma cells, causing cells and tumors to self-generate local gradients that motivate chemotaxis away from the tumor. LPA was the strongest serum-derived chemotactic motivator of several melanoma patient-derived cell lines (Muinonen-Martin et al., 2014). However, the *in vivo* significance of LPA-mediated chemotaxis or the generality of the importance of LPA in tumor dissemination is unknown. Here, we demonstrate an important role of LPA in PDAC cell chemotaxis and metastasis *in vivo*, mediated by N-WASP. Our study unexpectedly highlights N-WASP as a key driver of endosomal recycling of the major G-protein coupled receptor LPAR1which increases RhoA-mediated contractile responses and cell steering. We suggest this pathway as a potentially exciting future target against the dissemination of PDAC.

## Results

### N-WASP Deletion Enhances Mouse Survival of PDAC and Reduces Metastasis

**Figure 1:**
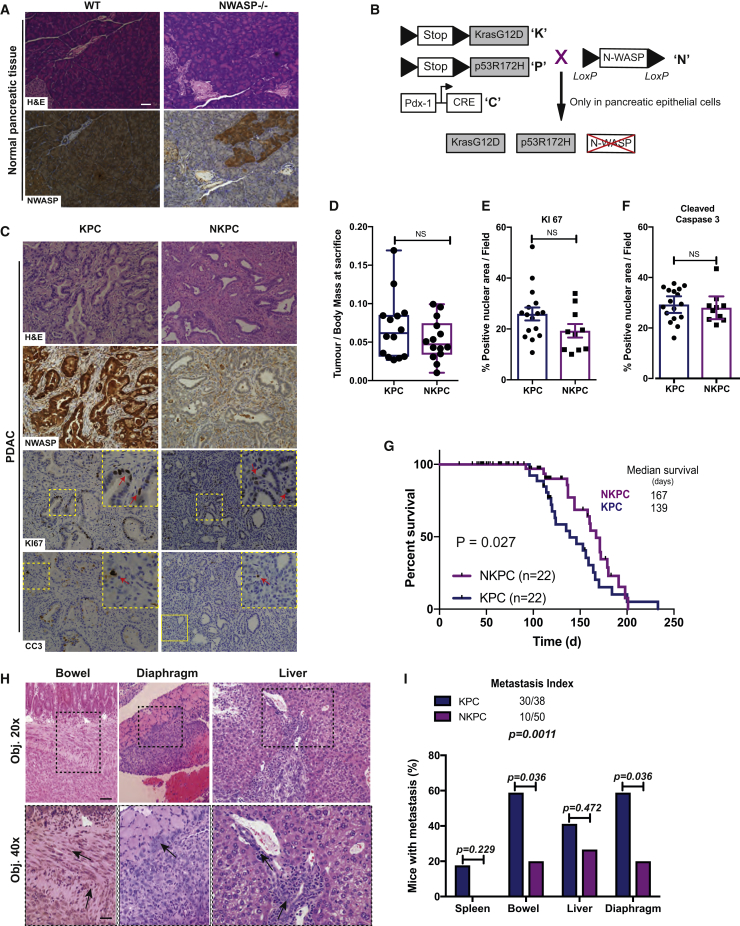
N-WASP Is Required for PDAC Dissemination (A) Representative haematoxylin and eosin (H&E) (top) and immunohistochemistry (N-WASP) (bottom) stained sections of normal pancreata. (B) Schematic representation of NKPC mice. (C) PDAC serial sections stained with H&E (top), N-WASP (middle top), KI67 (proliferation) (middle bottom), and cleaved caspase3 (CC3, apoptosis) (bottom). Inset panels are magnified areas of the yellow dashed box. Red arrows show positive cells. Scale bar, 50 μm. (D) Whisker plots showing tumor-to-body weight ratios at sacrifice (mean ± SEM; Unpaired t test, n = 14 KPC and 14 NKPC mice). (E and F) Quantification of KI67^+^ and CC3^+^ cells in PDAC from KPC and NKPC tumors (n > 10 fields/tumor from n ≥ 10 mice; mean ± SEM; Mann-Whitney test). (G) Survival curve (n = 22 KPC, 22 NKPC independent mice; Gehan-Breslow-Wilcoxon test). (H) H&E representative images of bowel, diaphragm, and liver metastasis of KPC mice. Arrows show the direction of collective invasion. Scale bars, 50 μm (upper panel) and 100 μm (lower panel). (I) Incidence and localization of KPC and NKPC metastasis (p values are from Fisher’s exact test). See also, Figure S1.

In human pancreatic cancer, high levels of N-WASP correlate with poor overall survival (Guo et al., 2014 and Figure S1A). Although N-WASP is expressed in normal pancreas, mosaic deletion of N-WASP using Pdx1::Cre had no effect on tissue structure, nor pancreatic functions (Figure 1A; Table 1). To further probe the mechanisms of N-WASP in promoting pancreatic cancer dissemination, we crossed N-WASP floxed mice into a model of genetically induced pancreatic ductal adenocarcinoma, the KPC (KRas p53 Cre) model (Hingorani et al., 2005). The model uses Kras^G12D^ and p53^R172H^, driven to express in the pancreas by Pdx-1::Cre (Figure 1B). Concomitant pancreas-specific deletion of N-WASP can also be driven by Pdx1::Cre (Figure 1B). Activation of the Pdx1::Cre initiates pancreatic neoplasia, developing into aggressive metastatic carcinoma with a half time of around 150 days (Hingorani et al., 2005). We refer to these mice and cell lines derived from them as KPC (KRas, p53, and Cre) and NKPC (N-WASP, KRas, p53, and Cre) throughout this study, and we compare NKPC with KPC mice and cells.

**Table 1:** Pancreatic Profile of Peripheral Blood from Mice in Cohorts Showing No Significant Changes upon Deletion of N-WASP in a Wild-Type or KPC Background

Mouse ID	Sex	Age (Days)	Glucose (mmol/L)	Urea (mmol/L)	Creatinine (μmol/L)	Triglyceride (mmol/L)	Amylase (U/L)	Lipase (U/L)
**NWASP**^**−**^**/**^**−**^								

147807	F	179	10.4	5.2	26	0.65	2,231	69
165057	F	193	17.5	5.9	29	0.9	1,761	57
165056	F	193	12.6	6.7	30	0.68	1,736	34
165054	F	193	15.5	6.8	32	0.78	2,115	41
165052	F	193	18.5	7.3	30	0.98	1,799	41
165047	M	191	14.4	5.6	28	0.83	2,753	65
150797	M	163	19.5	5.7	29	0.66	2,020	36
153701	M	147	14.5	7.5	28	0.94	2,465	46

**WT**								

156567	M	168	14.4	8.3	29	1.66	2,759	75
156572	M	168	14.4	6.9	29	1.11	2,481	69
170396	M	208	16.1	7.5	29	0.7	2,350	67
171667	F	206	16.9	5.6	30	0.67	2,077	64
176535	F	184	14	4.6	27	0.84	**3,455**	**475**
179782	M	163	19	6.1	26	0.76	**5,860**	**1,270**
152439	M	155	13.7	5.7	26	0.62	2,790	57
155587	M	138	9.7	6.9	26	0.53	**6,959**	**692**

Bold samples show abnormally high values of amylase and lipase, likely due to 24 h delay in analysis of blood samples.

At 15 weeks, mice showed neoplasia and PDAC, with a delay in lesion formation in NKPC mice (Figures S1B–S1D). Blood glucose and standard pancreatic function markers were normal (Figure S1E) at 15 weeks. By end-point, KPC tumors expressed N-WASP at very high levels (Figure 1C), displayed similar end-point histology to NKPC (Figures 1C and S1F–S1I), similar tumor mass (Figure 1D), and no significant difference in cell proliferation or death (Figures 1C, 1E, and 1F). NKPC tumors showed a higher CD31+ vessel density than KPC tumors (Figures S1I, middle panel and S1J), but comparable staining with Sirius red (collagen) (Figures S1I and S1K). However, mice with N-WASP-depleted tumors survived significantly longer prior to the onset of symptoms (Figure 1G), mainly due to the size of the primary tumor or accumulation of ascites fluid.

As this model is highly metastatic, we also evaluated the effect of N-WASP loss on PDAC dissemination. KPC tumors typically invade into the bowel adjacent to the primary tumor and metastasize to further invade the liver and diaphragm (Li et al., 2014 and Figures 1H and 1I). Local metastases (intestinal mesentery and spleen) and distant metastases (diaphragm, liver) were greatly reduced in NKPC mice (Figures 1H and 1I). Pancreatic cancer patients frequently present with ascites fluid in the peritoneal cavity, a feature also recapitulated in the KPC model. Strikingly, N-WASP deletion dramatically reduced the number of mice presenting with ascites and the volume of fluid (NKPC versus KPC, Figures S1L and S1M). Thus, loss of N-WASP delays the onset of PDAC in this model, but ultimately is dispensable for tumor growth and progression. However, N-WASP strongly contributes to metastatic potential in mice with PDAC.

### N-WASP Is Essential for KPC Cell Chemotaxis

**Figure 2:**
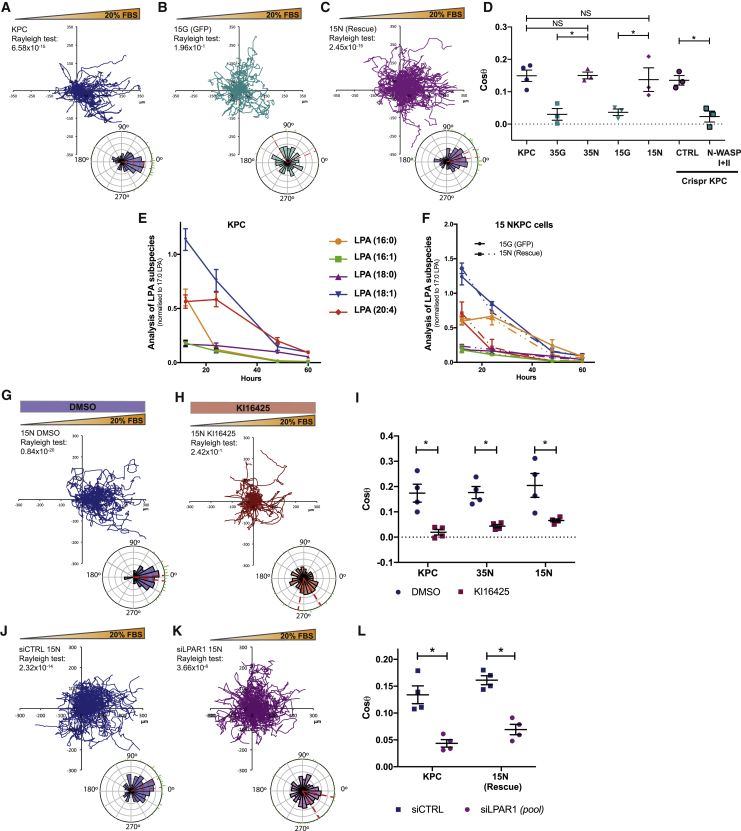
N-WASP Mediates PDAC Cell Chemotaxis to Serum LPA via LPAR1 (A–C) Representative cell tracks (Spider plots) and Rose plots with Rayleigh test for directionality (bottom circular graphs) for KPC (A), 15G NKPC (GFP) (B), and 15N NKPC (rescue) (C) migrating toward a 20% FBS gradient. (D) Scatter dot plot shows chemotactic index (Cosθ) from KPC, two different GFP expressing (35G and 15G) and GFP-N-WASP rescued (35N and 15N) as well as CTRL and N-WASP KPC CRISPR cells (as indicated). Mean ± SEM, n = 3, >150 cells per experiment (one-way ANOVA, ^∗^ ≤ 0.05, ^∗∗^p ≤ 0.01). (E and F) LPA subspecies analysis by mass spectrometry showing consumption over 60 h KPC PDAC cells (E) or 15 NKPC PDAC cells stably transfected with GFP (plain lines) or GFP-N-WASP (dotted lines) (F). Mean ± SEM, (n = 3 independent experiments). (G and H) GFP-N-WASP rescue NKPC cells with DMSO or 10 μM of KI16425 in 20% FBS gradient. Representative cell tracks (spider plot) and Rose plots with Rayleigh test for 15N NKPC (rescue) cells with treatments as indicated. (I) Scatter dot plots showing chemotactic index (Cosθ) from control (KPC) and N-WASP rescued (15N and 35N) cells. KI16425 abolished chemotaxis, mean ± SEM (n = 4 independent experiments, >150 cells/experiment, Mann-Whitney U test, ^∗^p ≤ 0.05). (J and K) LPAR1 knockdown inhibits pancreatic cancer cell chemotaxis toward 20% FBS gradient as shown when comparing spider plots, Rose plots, Rayleigh tests, and scatter plot of siRNA control (siCTRL) (J) versus a pool of siRNAs targeting LPAR1 (siLPAR1 pool) (K). (L) Scatter dot plot shows chemotactic index (Cosθ) from control (siCTRL) and LPAR1 knockdown (siLPAR1 pool) KPC and GFP-N-WASP rescued 15N [rescue] cells). Mean ± SEM, n = 4, >150 cells per experiment (Mann-Whitney test, ^∗^ p ≤ 0.05). See also Figures S2 and S3; Videos S1, S2, and S3.

A major mechanism for tumor dissemination is the migration of cancer cells from the primary tumor to/into blood vessels *in vivo*. To investigate the mechanisms by which N-WASP supports metastatic dissemination, we deleted N-WASP from KPC cells using CRISPR. We also generated independent cell lines from two different NKPC mouse tumors, which we rescued with GFP-N-WASP or labeled with GFP (Figures S2A and S2B). Using Insall chemotaxis chambers (Muinonen-Martin et al., 2014), we employed video microscopy and tracking to analyze the trajectories of the cells chemotaxing up a serum gradient. Spider plots showing the path taken by each cell and Rose plots summarizing the mean resultant vector of the migration show that KPC (Figures 2A and 2D; Video S1) and control CRISPR KPC (Figures 2D and S2C (iii); Video S1) cells were strongly chemotactic toward serum. All N-WASP-deficient cell lines showed a normal morphology during migration, but were defective in directional migration, including NKPC (Figures 2B and S2C(i); Video S1) or N-WASP CRISPR KPC cells (Figure S2C(iv); Video S1). Re-expression of N-WASP fully restored chemotaxis in NKPC cells (Figures 2C, 2D, and S2C (ii), d). Thus, N-WASP mediates the steering of PDAC cells up a gradient of serum, suggesting a role in chemotactic tumor cell dissemination.

Video S1. N-WASP is Required for PDAC Cell Chemotaxis, Related to Figure 2

### LPA Is the Driver of PDAC Cell Chemotaxis

Lysophosphatidic acid (LPA) is both a chemoattractant and a mitogen, present in a high level in body fluids, such as blood and ascites. LPA influences migration of pancreatic cancer cells *in vitro* (Komachi et al., 2009, Yamada et al., 2004). Melanoma cells and tumors break down LPA, generating a sink in regions of high cell density, leading to a self-generated chemoattractant gradient (Muinonen-Martin et al., 2014). Mass spectrometry analysis revealed that PDAC cells also rapidly metabolize LPA from serum in culture medium, and loss of N-WASP did not alter the rate of LPA consumption (Figures 2E, 2F, and S2E). However, N-WASP deficient tumor cells did not migrate toward a serum gradient. To probe the role of LPA in chemotaxis to serum, cells were treated with KI16425, an antagonist of the lysophosphatidic acid receptors LPAR1/3 (Ohta et al., 2003). N-WASP expressing cells were highly chemotactic toward serum (Figures 2G and 2I), but KI16425 treatment abrogated chemotaxis without affecting cell speed (Figures 2H, 2I, and S2F–S2H and Video S2). Similar results were obtained with the other cell lines (Figures 2I, S2F, and S2G; Video S2). RNA-sequence analysis (Figures S3A and S3B) combined with KI16425 specificity for LPAR1 and LPAR3 pointed to LPAR1 as the most likely receptor-mediating chemotaxis in KPC PDAC cells. To assess the connection with LPA and LPAR1 signaling in chemotaxis, we depleted LPAR1 by siRNA (Figures S3C and S3D) and demonstrated markedly reduced chemotaxic index, Cosθ, but little/no effect on cell speed or LPAR3 expression (Figures 2J–2L and S3E–S3G; Video S3). LPAR1 CRISPR KPC cell lines (Figure S3H) also showed severely reduced chemotaxis (Figures S3I–S3M; Video S3 but normal proliferation (Figure S3N). Thus, KPC cells rapidly consume LPA, creating a self-generated gradient, and both N-WASP and LPAR1 are crucial for chemotaxis of KPC pancreatic cancer cells toward serum LPA.

Video S2. LPA is the Driver of PDAC Cell Chemotaxis, Related to Figures 2 and S2

Video S3. LPAR1 is Crucial for Chemotaxis of Pancreatic Cancer Cells, Related to Figures 2 and S3

### N-WASP Influences the Balance between LPAR1 Degradation and Recycling

**Figure 3:**
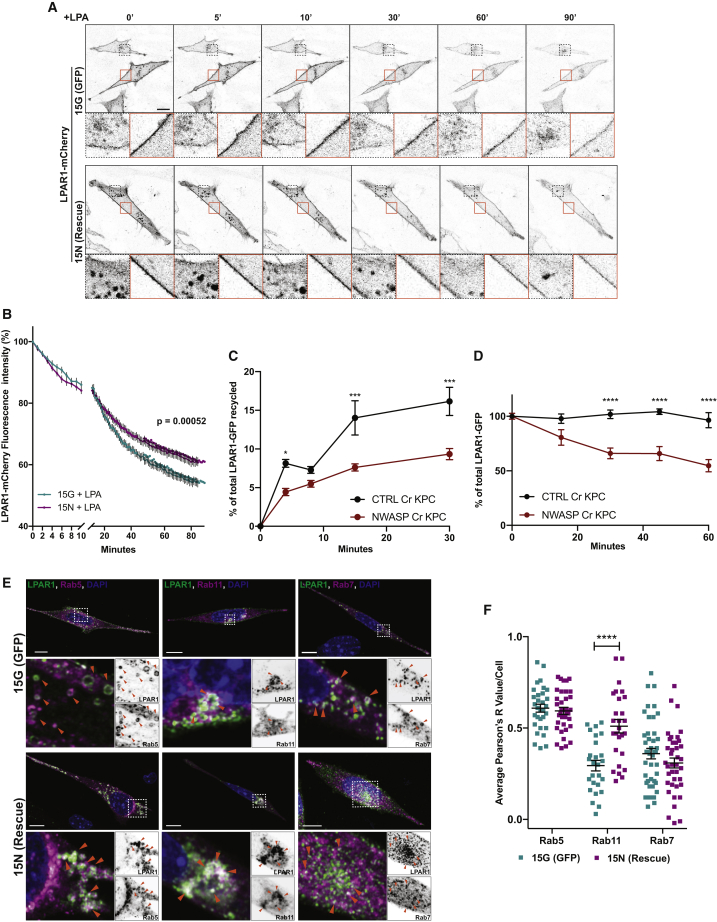
Loss of N-WASP Impairs LPAR1 Recycling (A) Inverted images of LPAR1-mCherry transfected 15G (GFP expressing, top panel) and 15N (GFP-N-WASP rescued, bottom panel) NKPC cells over time upon LPA stimulation. Confocal time-lapse movies 1 frame/min over 90 min. Stills from Video S4. Dashed black and plain orange insets are zoomed image of cytosol and plasma membrane, respectively. Scale bar, 20 μm. (B) Quantification of LPAR1 internalization upon LPA stimulation. Values are mean ± SEM, n = 4 independent experiments (>15 cells/experiment, t test, ^∗∗∗^ p ≤ 0.001). (C) CTRL and NWASP CRISPR KPC cells were transfected with LPAR1-GFP. Recycling of LPAR1-GFP was determined by capture-ELISA by looking at the quantity of biotinylated receptor recycled after 4, 8, 15, and 30 min after serum stimulation (30-min endocytosis). Values are mean ± SEM, n = 3 independent experiments (two-way ANOVA, Sidak’s multiple comparisons test, ^∗^p ≤ 0.05, ^∗∗∗^ p ≤ 0.001). (D) Degradation of LPAR1-GFP was determined by capture-ELISA. Graphs show the percentage of the receptors remaining at 0, 15, 30, 45, and 60 min after serum stimulation (30-min endocytosis). Data are mean ± SEM, n = 3 independent experiments (two-way ANOVA, Sidak’s multiple comparisons test, ^∗∗∗∗^ p ≤ 0.0001). (E) 15G and 15N NKPC cells were co-transfected with LPAR1-flag (green) and Rab5a-mCherry (magenta), after 5 min LPA stimulation (left panel) or co-transfected with LPAR1-flag (green), mCherry-Rab11a or Rab7-Rfp (magenta) (middle and left panel, respectively) at 2 h after stimulation with LPA. Orange arrowheads denote co-localization between LPAR1-flag and Rabs. Dapi, 4,6-diamino-2-phenylindole, in blue. Bottom panels show zoom of boxed images. Scale bar, 10 μm. (F) Co-localization between LPAR1-Flag and Rabs shown as Pearson’s R values in 15G (GFP) and 15N (rescue) NKPC cells. Bars show mean ± SEM, n = 3 independent experiments (>10 cells/experiment, t test with Welch’s correlation, ^∗∗∗∗^ p ≤ 0.0001). See also Figure S4, Video S4.

Given its association with actin and membranes, we speculated that N-WASP might regulate some aspect of LPAR1 trafficking to control chemotaxis. 7-transmembrane G-protein coupled receptors are rapidly internalized by endocytosis upon stimulation (Kang et al., 2014), and LPAR1 internalization depends on Rab5 (Murph et al., 2003). In unstimulated cells, LPAR1 was predominantly localized to the plasma membrane and was also visible within the endosomal compartments in the perinuclear region (Figure 3A, at 0 min, orange box and Video S4). LPA stimulation drove rapid internalization of LPAR1-mCherry (Figure 3A, at 5 to 90 min, orange box and Video S4). The rate of LPAR1-mCherry internalization was measured by tracking the fluorescence intensity at the plasma membrane over time and expressing this as a percentage of the total LPAR1-mCherry fluorescence at the membrane of each cell. Initial rates of LPAR1-mCherry internalization did not differ between N-WASP knockout cells (Figure 3B, 15G, cyan curve) and N-WASP rescued cells (Figure 3B, 15N, purple curve), and this was unaffected by addition of primaquine (PMQ) to inhibit receptor recycling (Figure S4A) (van Weert et al., 2000). However, at longer times and in the absence of PMQ, LPA stimulation led to a sharper decrease in cell surface LPAR1-mCherry in N-WASP knockout cells (Figure 3B). Co-localization analysis at 30 min post-stimulation revealed a modest increase in accumulated LPAR1 in the Rab7+ late endosome compartment (Figures S4B and S4C), supporting the idea that LPAR1 may increase traffic through this compartment and possibly be impaired in recycling in the N-WASP knockout cells.

Video S4. Loss of N-WASP Impairs LPAR1 Recycling to the Plasma Membrane, Related to Figure 3

To further interrogate a potential LPAR1 recycling defect following internalization, we used a quantitative biochemical approach to directly measure LPAR1 recycling by capture-ELISA (Roberts et al., 2001). LPAR1-GFP was rapidly recycled to the plasma membrane in CTRL CRISPR KPC cells, and this was significantly suppressed by CRISPR-mediated deletion of N-WASP (Figures 3C and S4D). A similar trend was observed for the transferrin receptor (TfnR) (Figure S4E). However, the total size of the internalized pool of LPAR1 was also robustly reduced in the N-WASP knockout versus control cells (Figure 3D), but less so for the transferrin receptor (Figure S4F), indicating that while recycling of both LPAR1 and TfnR is dependent on N-WASP, degradation of LPAR1 was specifically enhanced in N-WASP-depleted cells.

To get further insight into this process, we visualized LPAR1-FLAG together with markers of the early, recycling, and late endocytic compartments at various times after LPA addition. Upon LPA stimulation, LPAR1-FLAG moved from the plasma membrane to colocalize with Rab5 in early endosomes in both N-WASP null and rescued cells (Figures 3E, left panel and 3F) in line with the similar internalization rates observed in Figure 3B. However, subsequent to early endosome delivery, LPAR1-Flag followed different routes depending on the presence of N-WASP. Indeed, in N-WASP expressing cells, LPAR1-Flag moved to recycling endosomes, as was evidenced by significant co-localization of the receptor with Rab11 positive vesicles at 2 h following LPA addition (Figures 3E, middle panel and 3F). Also at 2 h, co-localization between LPAR1-flag with Rab7 was similar in N-WASP null and rescued cells, indicating that the increase seen at 30 min (Figures S4B and S4C) was transient and preceded degradation (Figure 3D). Taken together, these data indicate that N-WASP is required for trafficking of LPAR1 through recycling endosomes and back to the plasma membrane, and in the absence of N-WASP, internalized LPAR1 is trafficked to late endosomes and thence to lysosomes for degradation.

### N-WASP Drives LPAR1 Recycling via a SNX18 Compartment

**Figure 4:**
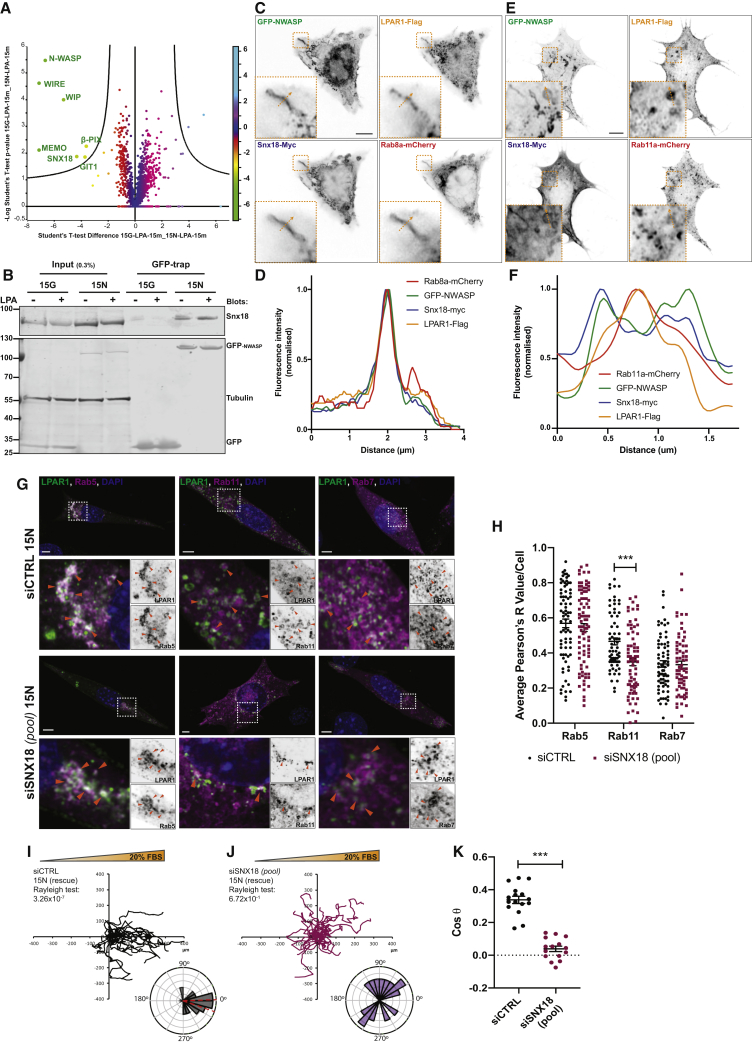
The N-WASP-SNX18 Complex Regulates LPAR1 Recycling and Chemotaxis in PDAC Cells (A) Volcano plot showing proteins binding to GFP-N-WASP in 15N (rescued cells) versus GFP in 15G (N-WASP knockout cells) after 15 min LPA stimulation (t test, p ≤ 0.05). Color scale indicates t test significance. (B) Snx18 co-immunoprecipitation with GFP-NWASP in 15N NKPC cells. Input and GFP-trap lysates were analyzed using anti-SNX18, anti-GFP, and tubulin as indicated. (C–F) 15N cells were transfected with LPAR1-Flag, Snx18-Myc, and Rab8a-mCherry (C) or mCherry-Rab11a (E) and stimulated 1h with LPA. LPAR1-Flag (yellow), Snx18-Myc (blue), N-WASP (green), and Rab8a-mCherry (red) colocalize on recycling tubule (C and D) or with mCherry-Rab11a (red) on Rab11a^+^ recycling vesicle (E and F) as shown on the fluorescence intensity profiles (orange dotted arrow). Insets show zoom. Scale bar, 5 μm. (G) siCTRL and siSNX18 (oligonucleotide pool) 15N NKPC cells were co-transfected with LPAR1-flag (green) and Rab5a-mCherry (magenta), after 15 min LPA stimulation (left panel), or co-transfected with LPAR1-flag (green), mCherry-Rab11a, or Rab7-Rfp (magenta) (middle and left panel, respectively) at 2h after stimulation with LPA. Orange arrowheads denote co-localization between LPAR1-flag and Rabs. Dapi, 4,6-diamino-2-phenylindole, in blue. Bottom panels show a zoom of boxed images. Scale bar, 5 μm. (H) Co-localization between LPAR1-Flag and Rabs shown as Pearson’s R values in siCTRL and siSNX18 15N (N-WASP rescue) NKPC cells. Bars show mean ± SEM, n = 3 independent experiments (>10 cells/experiment, t test with Welch’s correlation, ^∗∗∗^ p ≤ 0.001). (I and J) Representative spider plots and Rose plots with Rayleigh test for directionality, and scatter plot of 15N (rescue) NKPC cells transfected with siRNA control (siCTRL) (I) versus a pool of siRNAs targeting Snx18 (siSNX18 pool) (J) in the presence of 20% FBS gradient. (K) Scatter dot plot shows chemotactic index (Cosθ) from control (siCTRL) and Snx18 knockdown (siSNX18 pool) GFP-N-WASP rescued 15N (rescue) cells). Mean ± SEM, n = 4, >150 cells per experiment (Mann-Whitney test, ^∗∗∗^ p ≤ 0.0001). See also Figure S4; Video S5.

We next used an unbiased approach to investigate proteins interacting with GFP-N-WASP following LPA stimulation of PDAC cells. Aside from the main N-WASP interacting proteins, WIP and WIRE (García et al., 2016), two prominent hits were Beta-PIX and GIT1, which form a complex involved in regulation of G-protein coupled receptor internalization and signaling (Frank and Hansen, 2008). Snx18 was also identified and is implicated in tubulovesicular recycling endosomes (Figure 4A) (Håberg et al., 2008). We independently verified that N-WASP `bound endogenous SNX18 in PDAC cells (Figure 4B). To determine whether LPAR1 transits through a SNX18-dependent compartment, N-WASP cells (15N NKPC) were transiently transfected with Snx18-Myc, LPAR1-Flag, Rab8a-mCherry or mCherry-Rab11a, recycling endosome markers. LPAR1-Flag puncta appeared along GFP-N-WASP positive tubules emanating from the cell periphery (Figure 4C) and co-localized with Snx18-Myc and Rab8a-mCherry (Figures 4C and 4D). Similarly, LPAR1-Flag, GFP-N-WASP, and Snx18-Myc co-localized on mCherry-Rab11a positive vesicles (Figures 4E and 4F). Thus, N-WASP forms a complex with Snx18 on endocytic tubules mediating LPAR1 recycling and having strong implications for chemotactic signaling and cancer cell invasion.

To confirm this finding, SNX18-silenced N-WASP rescued cells (siSNX18 *(pool)* 15N) (Figures S4G–S4I) were transiently transfected with LPAR1-FLAG and a marker of early recycling or late endocytic compartment and stimulated with LPA 5 min or 2 h before fixation. Snx18-knockdown cells showed a similar accumulation of LPAR1-Flag in early endocytic Rab5-positive vesicles (Figure 4G, left panel and 4H). However, a significantly higher level of co-localization was detected between LPAR1 and recycling (Rab11 positive, Rab11^+^) vesicles in control-silenced 15-N cells (siCTRL 15N) at 2 h post-LPA stimulation in comparison to Snx18-knockdown (siSNX18 15N) cells (Figure 4G, middle panel, and 4 h). Co-localization between LPAR1-flag with Rab7 positive vesicles was similar, suggesting that SNX18 does not affect trafficking to late endosomes (Figure 4G, right panel, and 4 h). To validate a functional connection between SNX18 and LPAR1 recycling, chemotaxis assays were performed. SNX18-silenced N-WASP rescued cells showed markedly reduced chemotaxic index, Cosθ, but no effect on cell speed or SNX9 expression (Figures 4I–4K, S4I, and S4J; Video S5). Taken together, these data show that the N-WASP-SNX18 complex is essential for LPAR1 recycling and chemotaxis in pancreatic cancer cells.

Video S5. SNX18 is Important for PDAC Cell Chemotaxis, Related to Figure 4

### Loss of N-WASP Impairs RhoA Activation and Reduces Traction Forces in Pancreatic Cancer Cells

**Figure 5:**
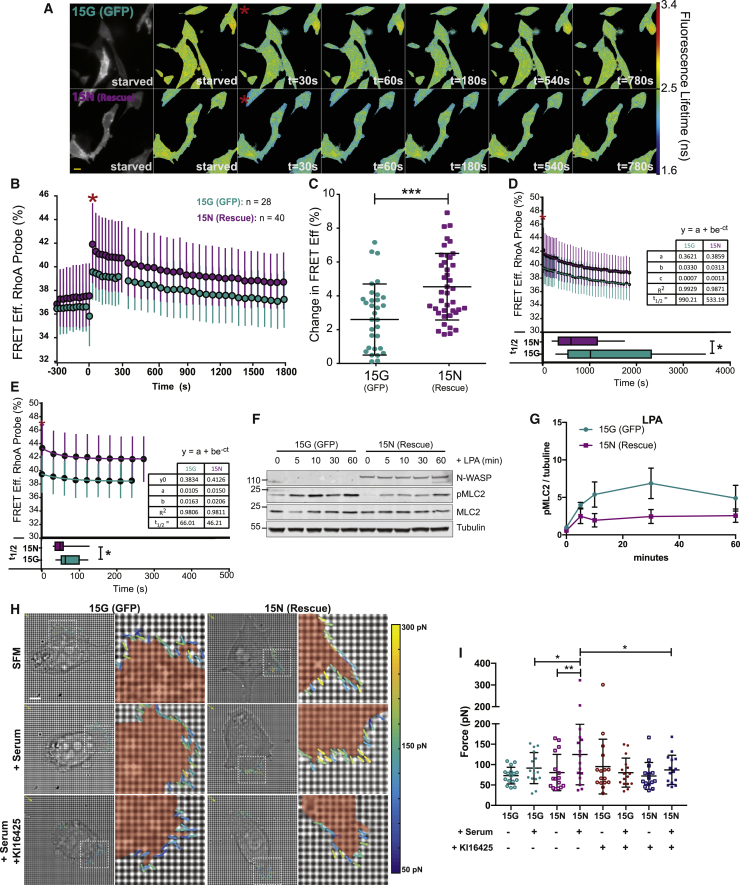
N-WASP Is Crucial for LPA-LPAR1-Mediated RhoA Activation and Traction Forces (A) Representative lifetime maps from Video S6 showing fluorescence lifetimes of the mTurquoise2 donor component of the RhoA-Raichu biosensor at times t. Red star denotes serum addition. (B) Percentage of RhoA-Raichu FRET efficiency after serum stimulation. Cyan and magenta circles indicate mean activity for 15G (GFP expressing) and 15N (GFP-N-WASP rescue) NKPC cells, respectively. Data are mean ± SD (n = 28 15G (GFP) cells and n = 40 15N (rescue) cells). (C) Change in FRET signal at 30 s relative to baseline (mean of times −300 to 0 s) in 15G and 15N cells. Whiskers represent mean ± SD, n = 3 independent experiments (n = 28 15G cells and n = 40 15N cells, t test, ^∗∗∗^ p ≤ 0.001). (D) Solid lines represent the fit of an exponential decay of the form y = a + be^−ct^ to the mean FRET efficiency data (circles) over 30 min post-stimulus. 15N data (magenta, n = 40); 15G data (cyan, n = 28). 30s time frame = zero point (maximum) of the decay. Error bars represent standard deviation. The box plot below the decay curve shows the distribution of half-times [t_1/2_ = ln(0.5)/( −c)] of the exponential fit to each individual data set (^∗^p ≤ 0.05). (E) As in (D), except over the first 5 min following stimulation. 15N data (magenta, n = 28); 15G data (cyan, n = 15). Box plot below shows the distribution of the half-times as above, (p < 0.05). (F) Western blot of phospho-MLC2 (Thr18/Ser19) after LPA stimulation as indicated showing also total MLC2 and tubulin. (G) MLC2 phosphorylation after LPA stimulation in 15G (cyan) and 15N (magenta) NKPC cells. Relative (Thr18/Ser19) MLC2 phosphorylation was normalized to tubulin. Graphs show mean ± SEM, 5 independent experiments. (H) Representative images of traction forces exerted by 15G (right column) and 15N (left column) before stimulation (top), after serum stimulation (middle), or in the presence of serum and KI16425 (bottom). (I) Quantification of traction forces generated by 15G (GFP) and 15N (rescue) NKPC cells. Bar graphs displays mean ± SEM measured across multiple pillars over 1.5 h (n = 16 cells/condition, paired t test, ^∗^ p ≤ 0.05, ^∗∗^ p ≤ 0.01). See also Figure S5; Video S6.

We predicted that the LPAR1 recycling defects caused by loss of N-WASP could impact on LPAR1 signaling and thus chemotactic migration. The strong link between LPA-LPAR1 and the RhoA GTPase prompted us to investigate the effects on RhoA-mediated traction forces. We used an optimized Raichu (Itoh et al., 2002) RhoA biosensor (Martin et al., 2018) to examine the dynamics of RhoA activity upon serum stimulation. Serum-starved rescue (15N NKPC) and the N-WASP-deleted (15G NKPC) cells displayed similar baseline RhoA biosensor FRET efficiency (Figures 5A and 5B, times previous to red stars and Video S6). Upon stimulation with serum, N-WASP rescued cells (15N NKPC) showed a larger increase in RhoA FRET efficiency (Figure 5C) than N-WASP knockouts. Furthermore, N-WASP rescued cells turned off RhoA activation more quickly, with a halftime of 533 s in comparison to 990s for N-WASP null cells (Figure 5D). When each cell is fitted with an independent decay, the data show a significant difference (p = 0.022) in the half-times between N-WASP rescue and N-WASP null cells. Furthermore, the early phase of the deactivation (0–300 s) fits more poorly than the later phase in the N-WASP rescue (15N NKPC) cells, suggesting a biphasic deactivation (Figures S5A and S5B). When the data between 0-300 s after stimulation were analyzed independently of the later time points, however, we saw the same trend—that the average N-WASP rescue cell showed a shorter halftime of decay in the measured FRET efficiency over time than the N-WASP null (15G NKPC) cells (Figure 5E). Indicating consequences of RhoA signaling, N-WASP knockout cells also showed an enhanced and sustained phosphorylation of myosin light chain MLC2 on (Thr)18 and (Ser19) (Figures 5F, 5G, S5C, and S5D). Concomitant with the alteration of myosin activation, traction forces exerted by N-WASP rescued (15N NKPC) cells increased after serum stimulation whereas N-WASP knockout (15G NKPC) cells exhibited no changes in traction force (Figures 5H, middle row and 5I; Video S7). Pre-treatment with the LPAR inhibitor KI16425 abrogated the contractile response, suggesting a predominant role of LPA and LPAR1 (Figures 5H, bottom row and 5I; Video S7). Thus, pancreatic cancer cells depend on LPAR1 signaling to activate a contractile response to LPA, likely crucial for coordinating motility and invasion. In summary, trafficking of LPAR1 mediated by N-WASP is crucial for the coupling of LPAR1 signaling with RhoA-mediated actomyosin contraction and force generation during tumor cell chemotaxis, invasion, and dissemination.

Video S6. Loss of N-WASP Impairs RhoA Activation in Pancreatic Cancer Cells, Related to Figure 5

Video S7. Loss of N-WASP Reduces Traction Forces in PDAC Cells, Related to Figure 5

### N-WASP Drives PDAC Invasion and Metastatic Seeding via a Chemotactic Loop with LPA-LPAR1

**Figure 6:**
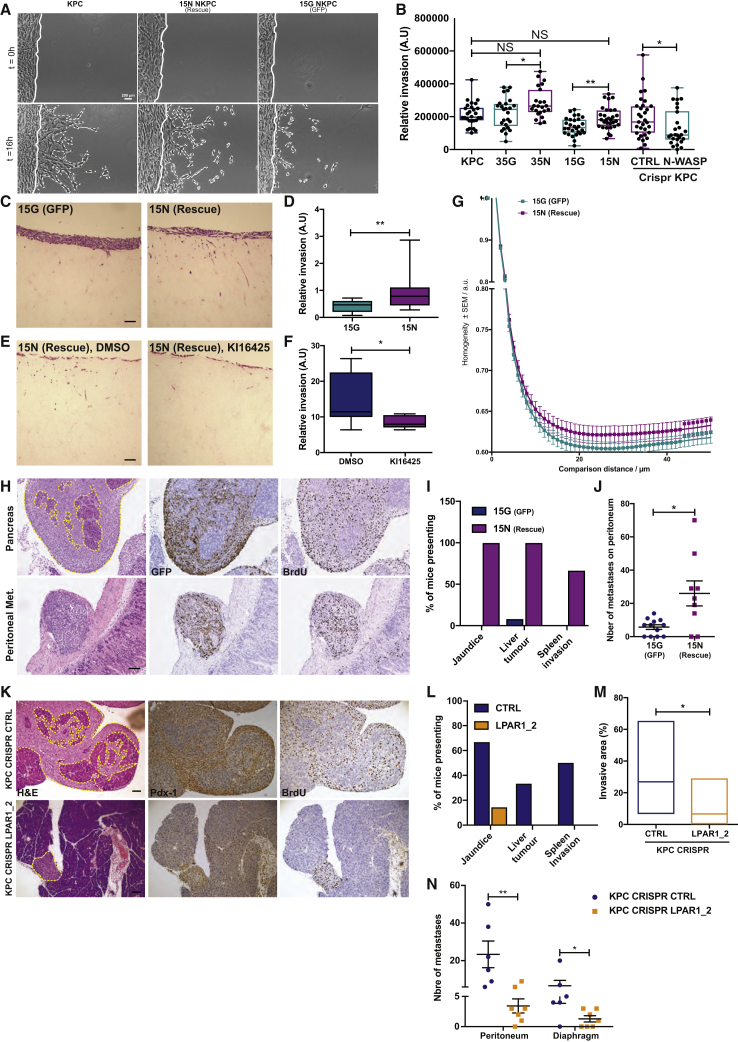
N-WASP Coordinates a Signaling Loop with LPA/LPAR1 Required for Force Generation Driving Invasion and Metastatic Seeding *In Vivo* (A) Invasion assay showing representative images of KPC and 15 NKPC cells stably transfected with GFP (15G) or rescued with GFP-N-WASP (15N) at t = 0 or 16 h after invading Matrigel. Scale bar, 200 μm. (B) Invasion index (see Figure S2A for nomenclature). Whisker plots showing all points; mean ± SEM, n = 4 independent experiments with >5 fields quantified each time (Mann-Whitney test or Welch’s t test were used to compare 15G versus 15N and CTRL versus N-WASP KPC CRISPR cells and 35G versus 35N, respectively: ^∗^ p ≤ 0.05, ^∗∗^ p ≤ 0.01). (C) Representative organotypic collagen invasion assay sections stained with H&E. Scale bar, 500 μm. (D) Quantification of relative invasive index (arbitrary units = A.U). Whisker shows 5^th^ and 95^th^ percentile (Mann-Whitney U test, n = 3 invasion assays per experiment, 3 independent experiments, and ^∗∗^p ≤ 0.01). (E and F) Representative images (E) and quantification (F) of 15N NKPC (rescue) cells invasion in collagen plugs treated with DMSO or KI16425. Whiskers show relative invasion (A.U.) mean ± SEM (Mann-Whitney U test, n = 3 organotypic invasion assays/experiment, 3–4 independent experiments, ^∗^p ≤ 0.05). (G) Gray-level correlation matrix texture analysis of angular second moment (ASM), a measure of SHG homogeneity (n = 4 independent experiments, each n = 10 fields of view). Mean of pixels ± SEM at each distance. (H) Representative H&E images of the pancreas (upper panel) and peritoneal metastasis (lower panel) invaded by 15N NKPC cells for (left), GFP (middle), and BrdU (right). Scale bar, 200 μm. (I) Incidence of mice presenting jaundice, liver tumor, and spleen invasion. (J) Number of metastases on peritoneum. n = 12 mice for GFP and n = 9 mice for GFP-NWASP. Mean ± SEM (Welch’s t test, ^∗^ p ≤ 0.05). (K) Representative images of H&E-, BrdU-, and Pdx-1-stained pancreatic invasion 15 days after intraperitoneal injection of control (CTRL, upper panel) or LPAR1 (lower panel) CRISPR KPC cells in nude mice. Scale bar, 200 μm. (L) Incidence of nude mice presenting jaundice, liver tumor, and spleen invasion when injected with control (CTRL, upper panel) or LPAR1 (lower panel) CRISPR KPC cells. (M) Quantification of the invasive area by CTRL or LPAR1 CRISPR KPC cells. Yellow dotted lines represent the limit of the invasive front. Floating bars represent the percentage of invasion normalized to the total surface of pancreas per section. Line is at mean (Mann-Whitney test, ^∗^p ≤ 0.05). (N) Number of metastases on the peritoneum and mean ± SEM (Mann-Whitney test, ^∗^p ≤ 0.05, ^∗∗^ p ≤ 0.01). (D–F) n = 6 mice for KPC CRISPR CTRL and n = 7 mice for KPC CRISPR PAR1_2. See also Figure S6.

Having established that N-WASP couples LPAR1 signaling to RhoA activation and contraction forces, we investigated how N-WASP and LPAR1 mediate invasion *in vitro* and metastatic seeding *in vivo*. As expected, N-WASP-depleted cells showed a significant reduction in invasiveness through Matrigel (Figures 6A, 6B, S6A and S6B; Video S8) in a circular invasion assay, which is dependent on both matrix remodeling and chemotaxis (Yu and Machesky, 2012). Furthermore, NKPC cells did not invade into collagen plugs previously remodeled by PDAC cancer-associated fibroblasts (CAFs) isolated from tumor-bearing KPC mice, while rescued cells showed significant invasion (Figures 6C and 6D). This assay was also previously shown to be heavily dependent on LPA-LPAR1 signaling, which we confirmed for N-WASP rescue cells (Figures 6E and 6F) (Muinonen-Martin et al., 2014). To examine whether coupling of LPAR1 to force generation for matrix remodeling was affected by loss of N-WASP, we cultured N-WASP-knockout and -rescued NKPC cells on top of freshly isolated peritoneum as a model for fibrillar collagen remodeling and degradation. Second Harmonic Generation (SHG) microscopy revealed a similar capacity of N-WASP knockout and rescued NKPC cells to degrade collagen fibers (Figure S6C). However, computational comparison of neighboring pixels of the z-stack maximum projections (Figures 6G and S6D, purple line) showed a more homogeneous distribution of collagen SHG signal in N-WASP re-expressing rescued NKPC than with NKPC cells (Figures 6G and S6D, cyan line), consistent with an inability of N-WASP knockout cells to generate force required to remodel the collagen. Therefore, N-WASP knockout PDAC cells not only show the inability to chemotactically steer through matrix in 3D invasion assays but also inability to generate force against micropillars or to remodel collagen in tissue explants.

Video S8. N-WAP is Required for PDAC Cells Invasion, Related to Figures 6 and S5

We next employed an assay to test metastatic seeding in the peritoneal cavity, requiring tumor cells to invade the mesothelial wall (Li et al., 2014) GFP and GFP-NWASP rescued NKPC cells were injected into the peritoneal cavity of nude mice, and both displayed a strong tropism toward the pancreas (Figure 6H, top). Strikingly, we observed that 100% of the mice injected with N-WASP rescued cells presented with jaundice and weight loss, two of the most common symptoms of pancreatic cancer (Figures 6I, S6E, and S6F). Moreover, mice injected with NKPC rescued cells displayed more liver tumors and spleen invasion (Figure 6I). Furthermore, intraperitoneal transplant of N-WASP-deficient NKPC cells resulted in significantly fewer metastatic foci on the mesentery (Figure 6J). These results are in line with our genetic model shown in Figure 1 and demonstrate that it is specifically N-WASP in the PDAC cells that mediates metastatic seeding *in vivo*. To determine whether metastatic seeding *in vivo* also depended on LPA-LPAR1, we transplanted LPAR1_2 CRISPR KPC cells to the peritoneal cavity and monitored seeding. A lower percentage of mice injected with LPAR1 knockout cells presented jaundice, liver tumor, and spleen invasion (Figures 6K and 6L). Furthermore, LPAR1_2 CRISPR KPC cells formed fewer and less invasive metastatic nodules on the peritoneum and diaphragm, confirming the LPA-LPAR1 axis as a major mediator of pancreatic cancer cell invasion and dissemination *in vivo* (Figures 6K, 6M, and 6N).

## Discussion

We have established that N-WASP is a crucial node in chemotactic receptor trafficking, driving invasion and metastasis of PDAC. Molecular insights into PDAC spread are urgently needed, as it is a disease with a dismal prognosis and very little progress has been made in treatment. A recent study correlated N-WASP with perineural invasion and poor prognosis in human PDAC (Guo et al., 2014), and our analysis of TGCA pancreatic ductal adenocarcinoma samples revealed a significant association of high N-WASP expression with poor survival (Figure S1A). Our study highlights a pathway involving N-WASP and the sorting nexin Snx18 as a trafficking hub for sorting LPAR1 away from degradation and promoting recycling back to the plasma membrane to foster directional cell migration during metastatic spread.

N-WASP clearly is important for matrix remodeling by pancreatic cancer cells, which agrees with previous studies in other cell types showing a role in invadopodia formation (Yu et al., 2012, Lorenz et al., 2004) and breast cancer metastasis (Gligorijevic et al., 2012). N-WASP drives cancer cell invasion *in vitro* (Hou et al., 2017, Gligorijevic et al., 2012, Yu et al., 2012) and is associated with progression and metastasis in mouse breast cancer models (Gligorijevic et al., 2012, Yu et al., 2012). However, we extend the mechanism by which N-WASP mediates remodeling to its role in the trafficking of LPAR1 to activate RhoA and myosin, resulting in coordinated contractile forces that can pull and align collagen fibers. A hallmark of PDAC is the accumulation of stiff desmoplastic stroma, which we propose may be driven in part by excessive LPA signaling leading to collagen remodeling. Receptor trafficking is a major regulator of signaling downstream of activation of G-protein coupled receptors, such as LPAR1, and our data suggest that LPAR1 can still signal to RhoA activation after internalization, but for this signaling to effectively couple RhoA activation with contractility, efficient receptor recycling is needed.

LPAR1 is a potentially exciting target for cancer metastasis. It has been previously explored as a target against idiopathic pulmonary fibrosis, and phase-II clinical trials were carried out on inhibitors developed by Bristol-Meyers Squibb (Budd and Qian, 2013). Mesenchymal stem cells have also been implicated in LPAR1-driven fibrosis (Lemos and Duffield, 2018). Preclinical trials in KPC mice using compounds such as BMS-986020 might reveal whether LPAR1 is a useful target against PDAC spread.

Human PDAC metastasizes to multiple sites, including liver and peritoneal cavity, frequently with ascites fluid (Fernández-del Castillo et al., 1995). Ascites fluid, as well as blood serum and other body fluids, are rich sources of LPA (Yamada et al., 2004), which is both a mitogen and chemoattractant (Valdés-Rives and González-Arenas, 2017). We recently implicated LPA as a major chemoattractant for melanoma cells and proposed that gradients of LPA are self-generated by tumors (Muinonen-Martin et al., 2014). N-WASP was implicated in chemotaxis of breast cancer cells toward EGF (Desmarais et al., 2009) and in macrophages (Zicha et al., 1998), but the mechanisms are unknown. Here, we show that N-WASP controls chemotaxis of PDAC cells to LPA gradients and likely acts as a major motivator of tumor cell egress from primary tumors and tropism toward metastatic sites, dependent on N-WASP. This involves the coupling of the LPAR signaling to RhoA mediating actin-myosin contractility and cell steering and also links to matrix remodeling.

While a role for N-WASP in endocytic recycling has not been previously described, it associates directly with SNX-Bar proteins, such as SNX9, via their SH3 domains interacting with N-WASP proline-rich domain (Park et al., 2010, Shin et al., 2008, Yarar et al., 2007). SNX9 shows homology to SNX18 and *in silico* analysis ( http://modpepint.informatik.uni-freiburg.de/ ) predicts SNX18 interaction sites in N-WASP. Our unbiassed analysis showed N-WASP associated with SNX18, GIT1, and β-PIX in KPC cells. GIT1 and β-PIX form a complex and are implicated in G-protein-coupled receptor trafficking (Frank and Hansen, 2008, Lahuna et al., 2005) and are known N-WASP interactors. SNX18 and SNX9 share function in endocytosis at the plasma membrane (Park et al., 2010), and we here implicate SNX18 in cooperation with N-WASP for LPAR1 rescue from degradation after internalization, leading to recycling back to the plasma membrane. Thus, SNX18 and N-WASP may be a hub for sorting away from degradation and recycling of LPAR1 and other receptors, similar to the role of WASH on early and late endocytic compartments (Carnell et al., 2011, Zech et al., 2011). Rab8 and Rab11 co-localized with N-WASP and SNX18 (this study), and these Rabs mediate recycling of integrins, transferrin receptor, and MHC1 (Hattula et al., 2006). We thus implicate receptor trafficking by SNX18, Rab8, Rab11, and N-WASP in the response of PDAC cells to the self-generated LPA gradient in tumors, driving metastasis.

Signaling via LPAR1 controls multiple pathways, leading to motility and cell growth (Yung et al., 2014). One of the crucial signaling pathways regulating motility downstream of LPAR1 is activation of the small GTPase RhoA, leading to coordination of contractile forces with the cytoskeleton to drive migration and remodeling of the extracellular matrix. The trafficking defect caused by loss of N-WASP led to inefficient RhoA activation downstream of LPAR1 and thus an uncoordinated contractile response. Given the importance of Rho-kinase in PDAC (Rath et al., 2017, Timpson et al., 2011), making a connection with N-WASP and LPAR1 may provide future avenues to address the desmoplastic stroma and fibrosis associated with PDAC aggressiveness.

## STAR★Methods

### Key Resources Table

**Table:** 

REAGENT or RESOURCE	SOURCE	IDENTIFIER
**Antibodies**		

N-WASP (30D10) Rabbit mAb antibody	Cell Signaling Technology	Cell Signaling Technology Cat# 4848; RRID:AB_10694415
Rabbit Anti-Human Ki67 (Ki-67) Monoclonal Antibody, Unconjugated, Clone SP6	Lab Vision	Lab Vision Cat# RM-9106-S; RRID:AB_149707
Cleaved Caspase-3 (Asp175) Antibody	Cell Signaling Technology	Cell Signaling Technology Cat# 9661; RRID:AB_2341188
Mouse Anti-Actin, Alpha-Smooth Muscle Monoclonal Antibody, Unconjugated, Clone 1A4	Sigma-Aldrich	Sigma-Aldrich Cat# A2547; RRID:AB_476701
CD31 Antibody	Abcam	Abcam Cat# ab28364; RRID:AB_726362
Monoclonal Anti-Alpha-Tubulin Antibody Produced in Mouse	Sigma-Aldrich	Sigma-Aldrich Cat# T6199; RRID:AB_477583
Mouse Anti-GFP Monoclonal Antibody, Unconjugated, Clone 4B10	Cell Signaling Technology	Cell Signaling Technology Cat# 2955; RRID:AB_1196614
SNX18 Polyclonal Antibody	Thermo Fisher Scientific	Thermo Fisher Scientific Cat# PA5-58113; RRID:AB_2647714
SNX9 Polyclonal Antibody	Thermo Fisher Scientific	Thermo Fisher Scientific Cat# PA5-56734; RRID:AB_2647734
DYKDDDDK Tag (9A3) Mouse mAb	Cell Signaling Technology	Cell Signaling Technology Cat# 8146; RRID:AB_10950495
DYKDDDDK Tag (D6W5B) Rabbit mAb	Cell Signaling Technology	Cell Signaling Technology Cat# 14793; RRID:AB_2572291
Mouse Anti-Myc-Tag Monoclonal Antibody, Unconjugated, Clone 9B11	Cell Signaling Technology	Cell Signaling Technology Cat# 2276; RRID:AB_331783
Anti-Myosin Light Chain 2 Antibody, Unconjugated	Cell Signaling Technology	Cell Signaling Technology Cat# 3672; RRID:AB_10692513
Phospho-Myosin Light Chain 2 (Thr18/Ser19) Antibody	Cell Signaling Technology	Cell Signaling Technology Cat# 3674; RRID:AB_2147464
Mouse Anti-GFP (Green Fluorescent Protein) Monoclonal Antibody, Unconjugated	Abcam	Abcam Cat# ab1218; RRID:AB_298911
CD71 Antibody	BD Biosciences	BD Biosciences Cat# 555534; RRID:AB_395918
Goat Anti-Mouse IgG (H&L) Secondary Antibody, DyLight 800 4× PEG	Thermo Fisher Scientific	Thermo Fisher Scientific Cat# SA5-35521; RRID:AB_2556774
Goat Anti-Rabbit IgG (H&L) Secondary Antibody, DyLight 800 4× PEG	Thermo Fisher Scientific	Thermo Fisher Scientific Cat# SA5-35571; RRID:AB_2556775
Donkey Anti-Mouse IgG (H+L) Highly Cross-Adsorbed Secondary Antibody, Alexa Fluor 680	Thermo Fisher Scientific	Thermo Fisher Scientific Cat# A10038; RRID:AB_2534014
Donkey Anti-Rabbit IgG (H+L) Highly Cross-Adsorbed Secondary Antibody, Alexa Fluor 680	Thermo Fisher Scientific	Thermo Fisher Scientific Cat# A10043; RRID:AB_2534018
Goat Anti-Mouse IgG (H+L) Secondary Antibody, Alexa Fluor 405	Thermo Fisher Scientific	Thermo Fisher Scientific Cat# A-31553; RRID:AB_221604)
Goat Anti-Rabbit IgG (H+L) Superclonal(TM) Secondary Antibody, Alexa Fluor 647	Thermo Fisher Scientific	Thermo Fisher Scientific Cat# A27040; RRID:AB_2536101
Goat Anti-Mouse IgG (H+L) Cross-Adsorbed Secondary Antibody, Alexa Fluor 647	Thermo Fisher Scientific	Thermo Fisher Scientific Cat# A-21235; RRID:AB_2535804
DAPI (4′,6-Diamidino-2-Phenylindole, Dihydrochloride) Antibody	Thermo Fisher Scientific	Thermo Fisher Scientific Cat# D1306; RRID:AB_2629482

**Bacterial and Virus Strains**		

lentiCRISPRv1-puro	Addgene	49535
psPAX2	Addgene	12260
pCMV-VSV-G	Addgene	8454

**Chemicals, Peptides, and Recombinant Proteins**		

LPA (Oleoyl-L α-Lysophosphatidic Acid Sodium Salt)	Sigma	L7260
DMSO	Fisher Chemical	15572393
KI16425	Cayman Chemicals	*10012659*
Primaquine	Sigma	160393
17:0 Lyso PA 1-heptadecanoyl-2-hydroxy-sn-glycero-3-phosphate (Sodium Salt)	Avanti Polar Lipids	857127
G-418 Solution	Formedium	G4185S
Hexadimethrine Bromide (Polybrene)	Sigma	H9268
HiPerFect Transfection Reagent	Qiagen	301707
Prolong Diamond Antifade Mountant	Invitrogen	P36965

**Critical Commercial Assays**		

PerfeCTa qPCR Real-Time PCR	QuantaBio	95139-012
RNAeasy Kit	Qiagen	#74104
Cell Line Nucleofactor™ Kit V	Lonza	VCA-1003
Calcium Phosphate Transfection Kit	Invitrogen	K2780-01
GFP-Trap Agarose	Chromotek	Gta-20
Precision Red Advance Protein Assay	Cytoskeleton, Inc.	#ADV02-B
Micro BCA Protein Assay Kit	Thermo Fisher Scientific	23235

**Deposited Data**		

Mass Spectrometry Proteomics Data	This paper	PXD014506

**Experimental Models: Cell Lines**		

Mouse KPC PDAC Cells (from mouse 127445, Male)	This paper	N/A
Mouse 15 NKPC PDAC Cells (from mouse 155275, Female)	This paper	N/A
Mouse 35 NKPC PDAC Cells (from mouse 35275, Female)	This paper	N/A

**Experimental Models: Organisms/Strains**		

Mouse: Pdx-1::Cre;Kras^G12D^;p53^R172H^ (KPC) Mice	Hingorani et al., 2003	N/A
Mouse: *N*-*Wasp*^fl/fl^	Cotta-de-Almeida et al., 2007	N/A
CD-1 Nude Mice		Charles River

**Oligonucleotides**		

LPAR3 Quantitec Primer	Qiagen	QT00264320
LPAR1 Quantitec Primer	Qiagen	QT00107709
Rn18s Quantitec Primer	Qiagen	QT02448075
Human LPAR1 cDNA	BioScience	IRATp970F0432D
LPAR1 FlexiTube Gene Solution Pool	Qiagen	Mm_LPAR1_2 SI04777703 ; Mm_LPAR1_1 SI04775491 ; Mm_Edg2_5 SI02670241
Snx18 FlexiTube Gene Solution Pool	Qiagen	Mm_Snag1_5 SI014398786, Mm_Snag1_3 SI01427433, Mm_Snag1_5 SI01427426, Mm_Snag1_1 SI01427419
All-Star Negative siRNA	Qiagen	SI03650318
PCR Primers, See Method Details	This paper	N/A

**Software and Algorithms**		

ImageJ	NIH	https://imagej.nih.gov/ij/
Prism 8	GraphPad	https://www.graphpad.com
Zen Black Zeiss	Zeiss	https://www.zeiss.com
Image Studio Lite Software	LI-COR	https://www.licor.com

### Lead Contact and Materials Availability

Further information and requests for resources and reagents may be directed to and will be fulfilled by the Lead Contact, Laura M. Machesky (l.machesky@beatson.gla.ac.uk).

### Experimental Model and Subject Details

#### Genetically Modified PDAC Mouse Model

All animal experiments were performed according to UK Home Office regulations and in consideration of Arrive Guidelines. *LSL-KRas*^*G12D*^, *LSL-p53*^*R172H*^, *Pdx1::CRE* (KPC) mice previously described in Hingorani et al. (Hingorani et al., 2003) were crossed with *N-WASP* flox mice (Snapper et al., 2001). Mice were genotyped by Transnetyx (Cordova, TN, USA).

#### Pancreatic Ductal Adenocarcinoma (PDAC) Cell Lines Isolated from Tumor-Bearing Mice

PDAC cell lines were generated from *LSL-KRas*^*G12D*^, *LSL-p53*^*R172H*^, *Pdx1::CRE* (KPC) or *N-WASP flox*, *LSL-KRas*^*G12D*^, *LSL-p53*^*R172H*^, *Pdx1-CRE* (NKPC) tumors as previously described (Li et al., 2014). PDAC cells were cultured in DMEM (Dubelcco’s modified Eagle medium) containing 5-g/l glucose, 10% fetal bovine serum (Gibco), 2mM glutamine (Gibco), and penicillin-streptomycin (Gibco) and verified for Pdx1 and p53 expression by Western blot.

### Method Details

#### Molecular Biology and Transfection

Rat GFP-N-WASP was a gift from Michael Way. Rab8a-mCherry was a gift from David Bryant and Snx18-myc from Sven Carlsson.

Human LPAR1 cDNA (IRATp970F0432D, Source Bioscience) was cloned into TOPO using LPAR1 Fw 5′-AAGCTTAGATCTCGAGGCCACCATG-3′ and LPAR1 Rv 5′-TGATCCACTAGTACTAACCACAGAGTGGTCATTGC-3′. In parallel, mCherry cDNA was cloned into TOPO using mCherry Fw 5′-AGTACTAGTGGATCAGTGAGCAAGGGCGAGGAG-3′ and mCherry Rv 5′-ATCGATGCGGCCGCTTACTTGTACAGCTCGTCCATG-3′. After sequencing, LPAR1-mCherry was fused using LPAR1 Fw and mCherry Rv. The fusion product was cloned into TOPO for sequencing, then sub-cloned into p-eGFP-N1 backbone using BglII and NotI. LPAR1-Flag plasmid was generated by PCR / restriction digest-based cloning from the LPAR1-mCherry plasmid using LPAR1-Flag Fw 5′-ACTGAAGGATCCATGGCTGCCATCTCTACTTCC-3′ and LPAR1-Flag Rv 5′-ACTGAAGCGGCCGCTTACTTGTCGTCATCGTCTTTGTAGTCAACCACAGAGTGGTCATTGCT-3′ (BamH1 and NotI sites).

LPAR1-GFP was generated by cloning Human LPAR1 cDNA into p-eGFP-N1 using LPAR1-GFP Fw 5′-CTCGAGGCCACCATGGCTGCCATCTCTACT-3′ and LPAR1-GFP Rv 5′-GGATCCGCAACCACAGAGTGGTCATTGCTG-3′.

PDAC mouse stably expressing GFP or GFP-tagged human N-WASP were generated using the Amaxa system (program A-033). GFP transfected cells were isolated using the BD Aria sorter Z6001 and cultured in media supplemented with 1mg/mL G418 (Formedium) to maintain the selection.

For transient transfections, the same Amaxa program was used. Following the manufacturers instructions, 1×10^6^ PDAC cells were transfected with 5μg of a combination of the following plasmids: Rab5-mCherry, Rab7-mRFP, Rab11a-mCherry, LPAR1-Flag, LPAR1-mCherry/LPAR1-GFP or Snx18-myc.

CRISPR guide RNAs guide were generated using the Optimized CRISPR design website (http://crispr.mit.edu/). Briefly, the gRNAs sequences with the highest score were cloned into lentiCRISPRv1-puro (Addgene, 49535).

mouse N-WASP_1: 5′-CACCGCACGTTGGTGACCCTCCGCG-3′,
mouse N-WASP_2: 5′-CACCGCCCGCGGAGGGTCACCAACG-3′,
mouse LPAR1_1: 5′-CACCGACGAATGAGCAACCGGCGCG-3′,
mouse LPAR1_2: 5′-CACCGAATGAGCAACCGGCGCGTGG-3′,
mouse LPAR1_3: 5′-CACCGGCCGGTTGCTCATTCGTGTA-3′,

2×10^6^ 293T cells were co-transfected with 10μg of pLentiCRISPR empty or containing the above gRNAs and the packaging plasmids (7.5μg of pSPAX2 (Addgene 8454) and 4μg of pVSVG (Addgene 12260) using the calcium phosphate method (Invitrogen). Two days later, the viral supernatants were filtered and used to infect 2×10^6^ PDAC cells in the presence of Polybrene (Sigma, H9268). 1μg/mL of puromycin was used to select transfected cells.

#### SiRNA Transfection

1×10^5^ PDAC cells were transfected with 20nM using HiPerFect Transfection Reagent (Qiagen) according to the manufacturer’s instructions and cultured for 72 h. The siRNAs targeting mouse LPAR1 were FlexiTube Gene Solution pool (Mm_LPAR1_2 SI04777703, Mm_LPAR1_1 SI04775491, and Mm_Edg2_5 SI02670241, Qiagen), the siRNA targeting SNX18 were FlexiTube Gene Solution pool (Mm_Snag1_5 SI014398786, Mm_Snag1_3 SI01427433, Mm_Snag1_5 SI01427426, Mm_Snag1_1 SI01427419). The non-targeting control was All-Star negative siRNA from Qiagen (SI03650318).

#### RNA Isolation and Quantitative PCR

Total RNA was extracted from PDAC cells using the RNAeasy kit (Qiagen, #74104) and RNA was quantified using the NanoDrop 2000c spectrophotometer (Thermo Scientific). 1μg of purified RNA was retro-transcribed using the QuantiTect Reverse Transcription kit (Qiagen). qPCR was performed using SYBR Green qPCR kit (Thermo Scientific) in a 7,500 Fast Real-Time PCR System (Thermo Fisher Scientific) with LPAR3 (QT00264320), LPAR1 (QT00107709) and Rn18s (QT02448075) Quantitec® Primers purchased from Qiagen. Rn18s was used as a reference gene. Experiments were performed in triplicate.

#### RNA Sequencing and Analysis

Quality of the RNA was assessed on an Agilent 2100 Bioanalyzer. The Illumina TruSeq RNA preparation kit v2.0 was used to prepare the RNA library. The RNA library was sequenced on the NextSeq 500 Platform using the High Output 75 cycles kit (2×36 cycles, paired-end reads, single index). Quality controls on the raw RNA-seq data were assessed by fastqc (http://www.bioinformatics.babraham.ac.uk/projects/fastqc/) and fastq_screen (http://www.bioinformatics.babraham.ac.uk/projects/fastq_screen/). RNA-seq reads were aligned to the mouse genome, GRCm38, using TopHat2 version 2.0.10 (Kim et al., 2013) with Bowtie version 2.1.0 (Langmead and Salzberg, 2012). Expression levels were determined and statistically analyzed by a combination of HTSeq version 05.4p3 (http://www-huber.embl.de/users/anders/HTSeq/doc/overview.html), the R 3.1.1 environment, utilizing packages from the Bioconductor data analysis suite and differential gene expression analysis based on a generalized linear model using the DESeq2 (Love et al., 2014).

#### PDAC Growth Assay

2×10^4^ PDAC cells were seeded into 6 well plates in triplicate. Cells were counted each day for 3 days using the cell counter. N=3 independent biological repeats.

#### Invasion Assays

Circular invasion assay was described previously in Yu and Machesky (Yu and Machesky, 2012). Briefly, a culture insert (Ibidi) was positioned in the middle of a 6-well glass-bottom dish, then, 6×10^5^ PDAC cells were seeded around and allowed to settle down and adhere overnight. The insert and media were removed, then 350μL of pure Matrigel was added and left to set for 45 min at 37°C. Finally, 2mL of complete media was added to the dish prior to imaging.

For organotypic collagen invasion assays, Cancer-Associated fibroblasts (CAFs) isolated from KPC tumors were embedded into rat tail collagen I and allowed to contract and remodel the collagen lattices during a week in the incubator. CAFs were the remove from the collagen discs using 10μg/mL of puromycin during 48 h. Collagen plugs were then washed three times with media. 6×10^6^ PDAC cells were then seeded on top of the collagen discs. Two days later, the collagen matrices were moved onto grids to generate the air-liquid interface. Complete DMEM or complete DMEM containing 10μM Ki16425 or DMSO was changed every three days. PDAC cells were allowed to invaded during 5 days. Collagen plugs were then fixed in 4% paraformaldehyde overnight, processed using histological staining and imaged.

#### Peritoneum Basement Membrane Assay and SHG Imaging

Fresh peritoneums were harvested from mice around 10 weeks old, mounted on 6.5-mm diameter Transwell chamber (BD), sealed with VALAP and soaked into 1-N ammonium hydroxide for 1 h. Membranes were then carefully washed with PBS 3 times and soaked overnight in PBS as described in Yu et al., 2012. The following day, 3×10^5^ PDAC cells were seeded and allowed to invade for 96 h. KPC cells were stained with Calcein-AM (Invitrogen) for 1h before imaging.

Collagen second harmonic images were collected using a LaVision BioTeC Trimscope II system equipped with a Coherent Chameleon Ultra II femtosecond pulsed laser. An excitation wavelength of 890 nm was used so that the SHG would be generated at a central wavelength of 445 nm and focused to the sample plane by a long working distance 20x (NA = 1.0) water immersion objective from Zeiss. A z-stack around 100 μm deep was imaged over a region of 500μm by 500μm, with at least three duplicates of each condition. Image analysis was performed using ImageJ. The UMB GLCM plugin (http://arken.nmbu.no//∼kkvaal/eamtexplorer/imagej_plugins.html) was used as the basis for the texture analysis but modified so as to run automatically through the four directions of comparison, for each of the 100 comparison distances. Firstly, the user selected a directory containing the collagen stack images. A maximum projection image was then produced and duplicated. The duplicate image was then automatically thresholded to produce a mask that was then applied to the original maximum projection image. This removed the background noise bias introduced in the GLCM analysis by selecting only the collagen SHG signal. The masked image was then passed to a modified GLCM texture. The output of the plugin for each image was 100 rows of the five texture parameters over each of four directions, so in total 2,000 parameter values. These were saved as a text data file for each image. When all the images in the directory were analyzed, the data files were processed using another ImageJ macro which output both the mean and individual values for each texture parameter for each image. These were then imported into Prism for plotting.

#### Chemotaxis Assay

1.5×10^5^ PDAC cells were plated on a fibronectin-coated glass coverslip then serum-starved overnight in serum-free DMEM containing 2mM glutamine as previously described in (Muinonen-Martin et al., 2014). Nikon TE2,000-E inverted time-lapse microscope equipped with a motorized stage (Prior), a Perfect Focus (PFS) and MetaMorph software. The Insall chambers were kept in a humidified Plexiglas box maintained at 37°C and 5%CO_2_. Images were taken every 30 min during 48 h. If treated with KI16425 or DMSO, cells were incubated for 1 h in media containing the inhibitor or vehicle before imaging.

Cells were manually tracked using the MtrackJ plugin (ImageJ). Only the cells present on the Insall chamber bridge at the beginning of the experiment were quantified. Cells were tracked until they moved off the bridge. Dying cells were excluded.

#### *In Vivo* PDAC Transplantation Assay

As previously described in Li et al. (Li et al., 2014), 1×10^6^ PDAC cells were washed 3 times in PBS and resuspended in 100μL of PBS were intraperitoneally injected into CD-1 nude mice (10-W old females, Charles River). Mice were sacrificed 2 weeks after injection.

#### Traction Force Microscopy

Elastomer based micropillar arrays were fabricated by mixing polydimethylsiloxane (PDMS; Sylgard 184, Dow Corning) base and curing agent at 10:1. A drop of the elastomer mix was placed on glass-bottom dishes (35-mm diameter, 0.17-mm thickness glass) and degassed. Silicon templates with submicron pits of 1.8-μm depth, 500-nm diameter, 1-μm center-to-center distance in a square array were molded on the elastomer mix. The elastomer mix was cured at 70°C for 12 h, resulting in pillar arrays with 2-MPa Young’s modulus and bending stiffness *k* = 3.16 nN/μm using the Euler-Bernoulli beam theory (Ghassemi et al., 2012). The silicon molds were removed from the elastomeric pillar arrays while completely immersed in 100% ethanol. After immersion in liquid and removal of silicon mold, the elastomeric pillar arrays need to be maintained in the fluid to prevent pillar collapse. To prepare pillar arrays for cell seeding, ethanol was replaced with phosphate buffered saline (PBS) while maintaining complete hydration. Afterwards, the pillar arrays were incubated with 20 μg/ml bovine fibronectin (Sigma Aldrich) for 1 h at 37°C. Prior to cell seeding, samples were washed thrice with PBS. Cells were then seeded at 3×10^5^ PDAC cells/dish with complete growth medium. Cells were allowed to attach to the pillar arrays for at least 3 h. After cell attachment, complete growth media was replaced with serum-free medium (SFM). Cells were serum starved for a maximum of 6 h before start of pillar and cell tracking. Cells and pillars were tracked using a 100× objective (numerical aperture 0.95, no immersion; Olympus) on a timelapse microscope (EVOS FL Auto 1 microscope, ThermoScientific) under standard cell culture conditions. Images of cells and pillars were taken at 2-minute intervals for 2 h. Cell medium was replaced with SFM, with or without KI16425 inhibitor prior to microscopy, as necessary. After cell tracking under SFM conditions, the medium was replaced with regular growth medium, with or without KI16425 inhibitor as necessary. At the end of tracking, cells were removed via trypsinization to obtain an image of the micropillars at the equilibrium position. Images out of focus were excluded from further analysis. Image stacks of cells across time were drift corrected using the MultiStackReg plugin for ImageJ (National Institutes of Health). Pillar displacement from the equilibrium position was measured through cross-correlation of pillar pixel intensity (Gelles et al., 1988). To obtain cell traction forces, pillar displacement was obtained by multiplying displacements with the micropillar bending stiffness. Three independent experiments were performed with at least 5 cells tracked per experiment. Each data point is the mean cell traction force exerted by one cell on 20 pillars across at least 1.5 h.

#### LPA Extraction and Analysis by Liquid Chromatography-Mass Spectrometry

5×10^5^ PDAC cells were plated in a 6-well plate with medium containing 10% serum. Media samples were harvested every 12 h for 96 h. Prior to butanol extraction, 0.5μg of synthetic 17:0 LPA (Avanti) was added to samples. Liquid chromatography-mass spectrometry analysis was based on Susanto et al. (2017). Lysophosphatidic acids (LPAs) were analyzed using a Q-Exactive Orbitrap mass spectrometer coupled to an UltiMate 3,000 LC system (Thermo Scientific). The LC parameters were as follows: 5 μl of the sample was injected onto a 100×2.1 mm, 1.7-μm Waters ACQUITY CSH C18 column, which was kept at 50°C throughout the analysis. A gradient system of (A) aqueous 0.05% ammonium hydroxide and (B) 0.05% ammonium hydroxide/methanol was used, with a linear gradient of 0.3 ml/min from 50% to 90% B over 7 min rising to 100% B within 1 min and maintained for 2 min. Thereafter, the column was returned to initial conditions and equilibrated for another 4 min. LPAs were analyzed in electrospray negative ionization mode, at a resolution of 70,000, using a scan range of 300–700 m/z. LPAs were identified by the exact mass of the negatively charged ion (mass accuracy below 5ppm) and by known retention time on the LC column (from analysis of commercial LPA standards). Peak areas for each LPA were determined using TraceFinder (Thermo Scientific) and normalized to the peak area of the LPA (17:0) internal standard.

#### LPAR1-mCherry Internalization Dynamics

15G and 15N NKPC cell lines were transfected with LPAR1-mCherry plasmid as described above, plated on fibronectin-coated 6-well plate, serum-starved overnight and imaged at 24 h. Live-cell imaging used a confocal Nikon A1R Z6005 by using a Plan Fluor 63×/1.30 Oil objective at 37°C/5%CO2. Cells were imaged for 90 min with 1min frame intervals using 561-nm laser. ImageJ was use quantify LPAR1-mCherry fluorescence intensity at the cell membrane.

#### Recycling, Degradation Assay, and Capture-ELISA

CTRL and N-WASP CRISPR KPC cells transiently transfected with LPAR1-GFP were grown to achieve a confluence of 70%–80% before conducting receptor recycling or degradation assays. Celle were placed on ice, incubated in serum-free DMEM for 4 h. Cells were washed twice in cold PBS then surface-labeled with sulpho-NHS-SS-biotin (0.13mg/mL, Pierce) for 30 min at 4°C. Then, cells were allowed to internalize receptors in DMEM supplemented with 10% serum for 30 min at 37°C. The biotin from the protein remaining at the cell surface was reduced with 20mM mesna for one hour at 4°C, followed by quenching with 20mM iodoacetamide for 10 min at 4°C. The internalized fraction was then chased for the indicated time by returning the cells at 37°C, followed by a second MesNa treatment to cleave the biotin from the recycled receptors. Biotinylated LPAR1-GFP and transferrin receptors were determined by capture-ELISA using Maxisorp (Nunc) plates coated with the following antibodies GFP (Abcam, ab1218) or anti-CD71 for TfnR (BD Pharmingen 555534).

#### Fluorescent Lifetime Imaging

Frequency domain lifetime images were acquired at 60× magnification on a Nikon TE2000 microscope equipped with the Lambert Instruments Fluorescence attachment system, modulating the excitation light and the sensitivity of the intensifier at a frequency of 40MHz. The system functions with a lifetime resolution of <100ps, according to the manufacturer’s specifications. An LED emitting at wavelength 445nm was used to excite the FRET Donor mTurquoise2. Reference measurements where made from fluorescein (τ = 4.000 ns).

For time courses the software was set to take a FLIM image every 30 seconds for 5 min (early phase), and then one image every minute for 30 min (late phase). Late phase imaging was begun by the operator 60s after early phase completed. The Nikon Perfect Focus System was used throughout to prevent Z-drift. Lifetime information from individual cells was extracted using the tools available in the accompanying LI-FLIM software.

#### FRET Efficiency Calculations, Curve Fitting, and Statistical Analysis

Measured donor lifetimes were converted into FRET Efficiency using the equation E = 1-(τ_sensor_/τ_donor_). Donor lifetime was measured over-time under the same conditions as the biosensor was imaged, allowing each experimental time point to be normalized to its equivalent control time point. Calculations and transformation were carried out using Microsoft Excel. Plots, fits, and statistical analyses were performed in Sigma Plot 11.0.

#### Immunofluorescence

PDAC cells were fixed with 4% PFA for 15 min, then permeabilized with 0.2% Triton X-100 in PBS for 5 min, blocked for 5 min in 4% BSA in PBS. Primary antibodies were incubated 1 h in 4% BSA in PBS and detected with species-specific Alexa405, Alexa488, Alexa568 and Alexa647-conjugated secondary antibodies (Life Technologies). DAPI (Sigma) was used to stain nuclei. Coverslips were then mounted with Prolong Diamond Antifade Mountant (Invitrogen). Cells were imaged with a Zeiss 880 Laser Scanning Microscope with Airyscan with a Plan-Apochromat 63×/1.4 oil DIC M27 objective.

For colocalization analysis, the method was Adapted from Tyrrell et al., 2016. The ImageJ function “Subtract Background” was used on the LPAR1 or Rab5/Rab11a/Rab7 channels. Then, a 15×15 μm box was placed over the center of the cell. The selected ROI was then analyzed using the Coloc2 plugin from ImageJ.

#### GFP Trap and Mass Spectrometry Analysis

Cells were stimulated or not with LPA (sigma) during 15 or 60 min, collected in ice-cold lysis buffer (100mM NaCl, 25mM Tris-HCl pH7.5, 5mM MgCl2, 0.5% NP-40 supplemented with protease and phosphatase inhibitors (Pierce) and centrifuged at 4°C for 15 min. 1.5mg of protein were mixed with pre-equilibrated beads (Chromotek, GFP-Trap®) following the manufacturer instructions and incubated 2 h at 4°C. Purified proteins from triplicate biological replicates were digested with Lys-C (Alpha Laboratories) and trypsin (Promega) on beads as previously described (Hubner et al., 2010). Tryptic peptides were separated by nanoscale C18 reverse-phase liquid chromatography using an EASY-nLC 1,200 (Thermo Fisher Scientific) coupled online to an Orbitrap Q-Exactive HF mass spectrometer (Thermo Fisher Scientific) via nanoelectrospray ion source (Thermo Fisher Scientific). Peptides were separated on a 20 cm fused silica emitter (New Objective) packed in house with reverse-phase reprosil Pur Basic 1.9 μm (Dr. Maisch GmbH). For the full scan a resolution of 60,000 at 250th was used. The top ten most intense ions in the full MS were isolated for fragmentation with a target of 50,000 ions at a resolution of 15,000 at 250th. MS data were acquired using the Xcalibur software (Thermo Fisher Scientific).

The MS Raw files were processed with MaxQuant software (Cox and Mann, 2008) version 1.5.5.1 and searched with Andromeda search engine (Cox et al., 2011), querying UniProt (UniProt Consortium, 2010) *Mus musculus* (20/06/2016; 57,258 entries). The database was searched requiring specificity for trypsin cleavage and allowing maximum of two missed cleavages. Methionine oxidation and N-terminal acetylation were specified as variable modifications and cysteine carbamidomethylation as fixed a modification. The peptide, protein, and site false discovery rate (FDR) was set to 1 %. The common reverse and contaminant hits (as defined in MaxQuant output) were removed. Only protein groups identified with at least one uniquely assigned peptide were used for the analysis. For label-free quantification, proteins quantified in all 3 replicates in at least one group, were measured according to the label-free quantification algorithm available in MaxQuant (Cox et al., 2014). Significantly enriched proteins were selected using a t test analysis with a 5% FDR.

The raw files and the MaxQuant search results files have been deposited as partial submission to the ProteomeXchange Consortium via the PRIDE partner (Perez-Riverol et al., 2019) with the dataset identifier PXD014506.

#### Western Blotting

Cell were lysed for 5 min on ice in RIPA buffer (10 mM Tris-HCl pH7.5, 150 mM NaCl, 1mM EDTA, 1% Triton-×100, 0.1% SDS) supplemented with Halt protease inhibitor cocktail and Halt phosphatase inhibitor cocktail (Pierce), then centrifuged for 15 min at 4°C. Protein concentration was determined using Precision Red (Cytoskeleton)

For phospho-MLC2 western blots, cell lysates were prepared using cell lysis buffer (1%SDS, 50mM Tris ph7.5) and the protein concentration was measured using microBCA Protein assay kit (Thermo Scientific).

Samples were analyzed by SDS-PAGE using NuPage Novex (San Diego, United States of America) 4%–12% bis Tris gels and transferred on a nitrocellulose membrane. Secondary antibodies were detected using LiCor Odyssey.

#### Immunohistochemistry

IHC was performed on formalin-fixed paraffin-embedded sections using standard protocols. For collagen staining, sections were rehydrated and then immersed in Picro Sirius red solution (0.1% Direct red 80, Sigma 41496LH and 0.1% Fast green FCF, Raymond Lamb S142-2) for 2 h.

Slides were imaged using the Leica SCN 400f scanner and analyzed using Leica Slidepath Digital Hub software.

### Quantification and Statistical Analysis

Data analyses were done using GraphPad Prism 8. Statistical tests used are indicated in each figure legend. p values < 0.05 were considered as significant: ^∗^: p ≤ 0.05, ^∗∗^: p ≤ 0.01, ^∗∗∗^: p ≤ 0.001.

### Data and Code Availability

The proteome data have been deposited on the EMBL-EBI Pride Archive. The data set identifier is: PXD014506.

## Supplemental Information

Document S1. Figures S1–S6

Document S2. Article plus Supplemental Information
