# Supplementary material for: N-WASP Control of LPAR1 Trafficking Establishes Response to Self-Generated LPA Gradients to Promote Pancreatic Cancer Cell Metastasis
Source: Dev Cell. 2019 Nov 18;51(4):431–445.e7. doi: 10.1016/j.devcel.2019.09.018 (PMC6863394; doi:10.1016/j.devcel.2019.09.018)
Supplement: Document S1. Figures S1–S6 [file mmc1.pdf]

Developmental Cell, Volume *51*

Supplemental Information

N-WASP Control of LPAR1 Trafficking Establishes Response to Self-Generated LPA Gradients to Promote Pancreatic Cancer Cell Metastasis

Amelie Juin, Heather J. Spence, Kirsty J. Martin, Ewan McGhee, Matthew Neilson, Marie F.A. Cutiongco, Nikolaj Gadegaard, Gillian Mackay, Loic Fort, Sergio Lilla, Gabriela Kalna, Peter Thomason, Yvette W.H. Koh, Jim C. Norman, Robert H. Insall, and Laura M. Machesky

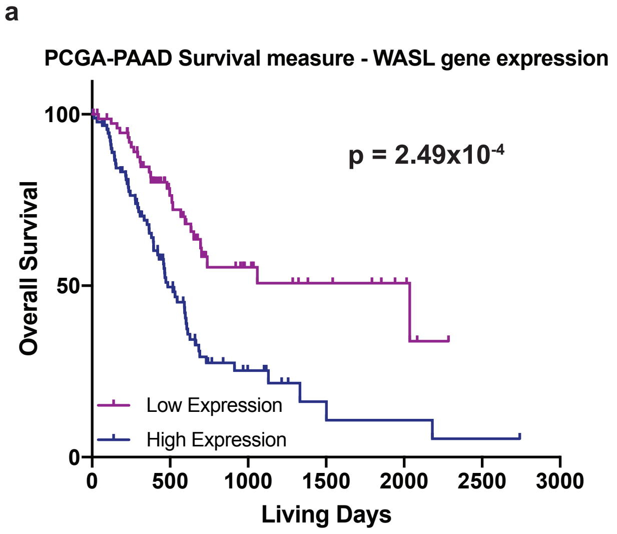

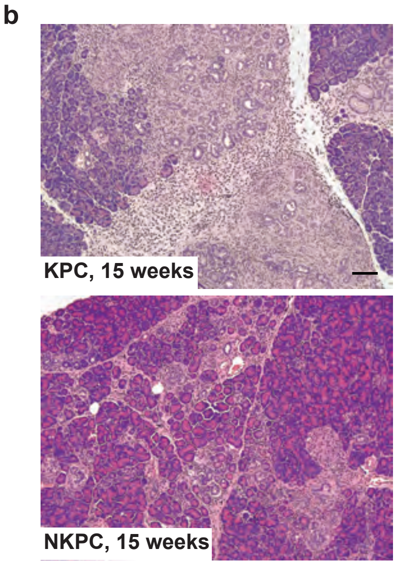

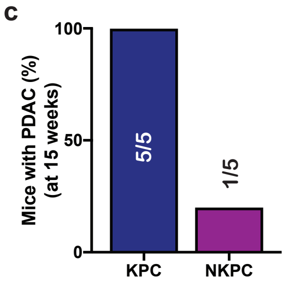

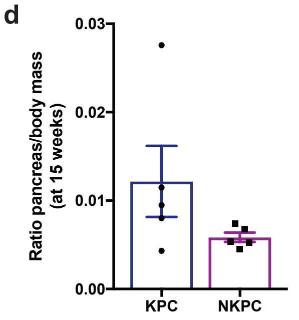

e

NKPC				Pancreatic Profile				
	Sex	Age	Glucose	Urea	Creatinine	Triglyceride	Amylase	Lipase
Mouse ID		(Days)	(mmol/L)	(mmol/L)	(μmol/L)	(mmol/L)	(U/L)	(U/L)
141740	M	87	21.4	7.3	31	0.72	2422	51
143117	M	77	30.8	6.6	34	0.59	1891	27
35.1f	F	104	N/A	9.7	37	0.64	2152	46
43.1c	M	104	26.8	8.7	32	0.84	2128	48

KPC				Pancreatic Profile				
	Sex	Age	Glucose	Urea	Creatinine	Triglyceride	Amylase	Lipase
Mouse ID		(Days)	(mmol/L)	(mmol/L)	(μmol/L)	(mmol/L)	(U/L)	(U/L)
11.2a	M	105	18.5	10.5	36	0.6	2263	35
11.2g	F	105	18.3	7.4	28	1.01	2308	48
18.2b	F	103	15.4	6.3	30	0.71	2883	105
18.2d	F	103	18.3	6.9	33	1.02	5336	340
18.2e	F	103	20.1	7.7	33	0.84	2870	98

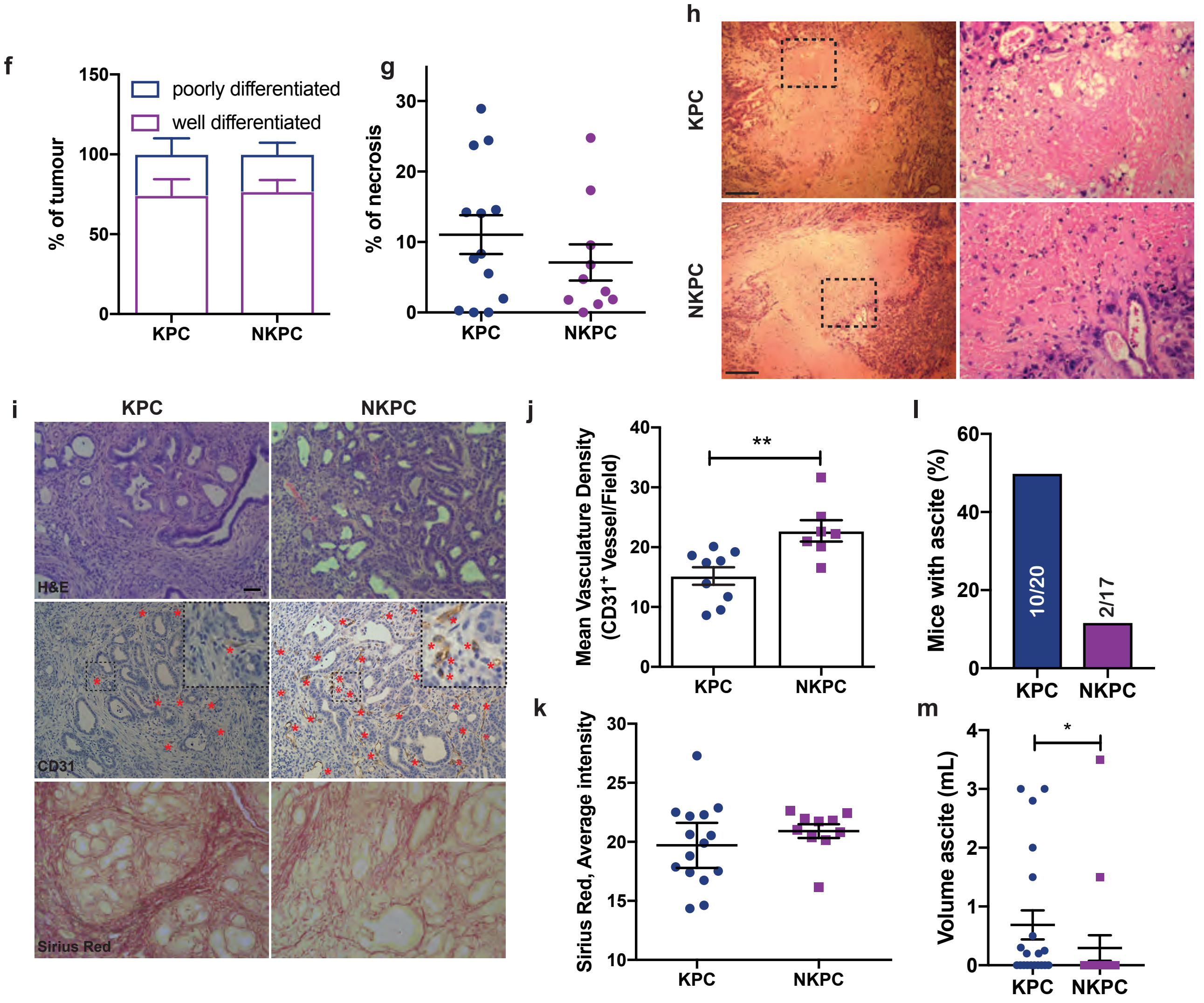

Figure S1

Figure S1 (related to Figure 1): Loss of N-WASP enhances survival and generates minor histological differences. (a) Kaplan-Meier curve showing the cumulative overall survival of patients in the TCGA cohort for high (blue, n = 85 patients) and low (purple, n = 85 patients) Wasl gene expression from Human Protein Atlas www.proteinatlas.org [1] (p = 2.49x10^{-4}, log-rank). (b) Representative H&E of 15 weeks KPC (*top panel*) and NKPC (*bottom panel*) pancreata. Scale bar = 500µm. (c) Incidence of PDAC in 15 week-KPC and -NKPC mice (n=5 KPC and n=5 NKPC mice). (d) Bar graph showing tumour-to-body weight ratios at 15 weeks (n=5 KPC and n=5 NKPC mice). (e) Blood results of pancreatic function of 15 weeks old KPC and NKPC mice (n=5 KPC and n=4 NKPC mice). (f) Grading of KPC and NKPC tumours. Bar graph shows percentage of tumour presenting well *vs* poorly differentiated PDAC lesions. Mean ± SEM (n = 18 mice KPC and NKPC independent tumours). (g-h) Percentage of necrotic areas (g) and representative H&E staining in KPC and NKPC tumours (h). Mean ± SEM (n = 13 KPC and n = 11 NKPC independent tumours, unpaired t-test, Not Significant). Scale bar = 200µm. (i) Representative serial sections of H&E, CD31 and Sirius red staining of KPC and NKPC tumours. Scale bar = 500µm. (j) Scatter plots showing mean vasculature density. Mean ± SEM (n = 9 KPC and n = 7 NKPC independent tumours, >5 fields of 20µm^2 quantified/tumour, Mann-Whitney test, **p≤0.01). (k) Bar graph showing Sirius Red average intensity normalized to total pancreatic area (n = 15 KPC and n = 10 NKPC tumours, Mann-Whitney t-test, non-significant). (l) Incidence of ascites in tumour-bearing mice (n = 20 KPC and n = 17 NKPC mice). (m) Volume of ascites. Mean ± SEM (n = 20 KPC and n = 17 NKPC mice, exact Wilcoxon rank sum test, *p≤0.05).

a

Cell type	Nomenclature
KPC cells	KPC
Line 35 NKPC cells expressing GFP	35G
Line 35 NKPC cells expressing GFP-N-WASP	35N
Line 15 NKPC cells expressing GFP	15G
Line 15 NKPC cells expressing GFP-N-WASP	15N
KPC with CRISPR CTRL	CTRL CRISPR KPC
KPC with NWASP CRISPR Knockout	NWASP CRISPR KPC

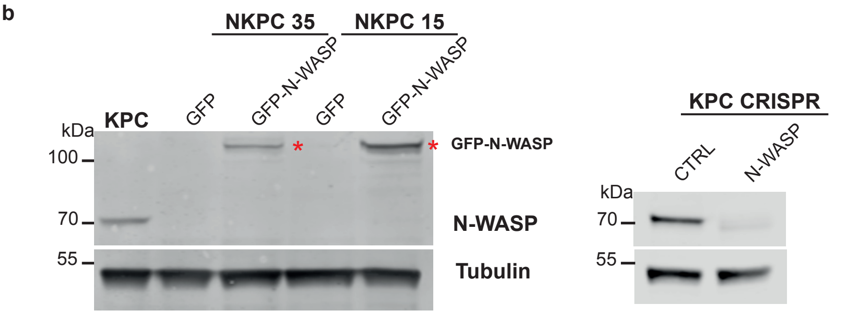

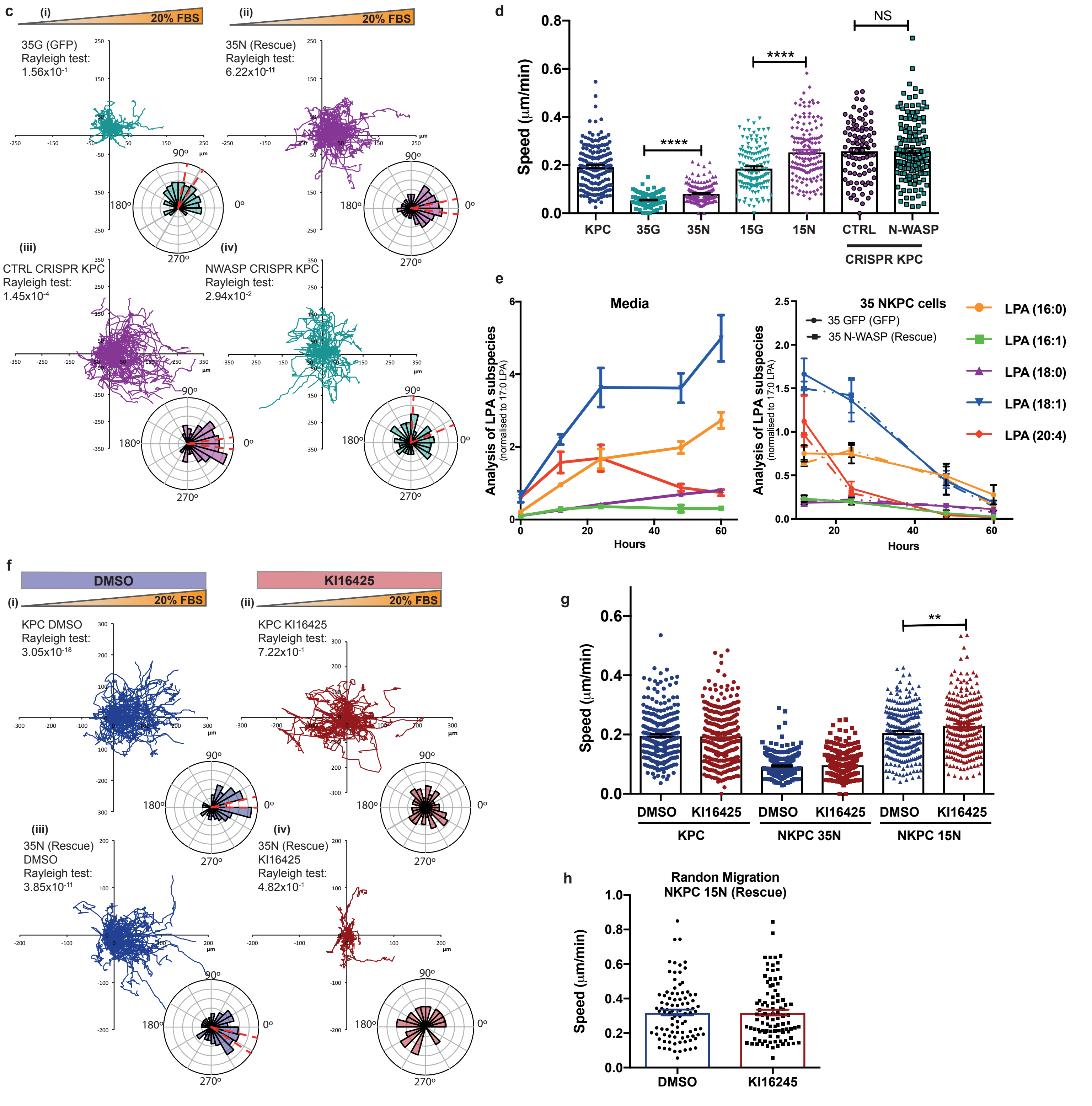

Suppl. Figure 2

Figure S2 (related to Figure 2): Loss of N-WASP impairs chemotaxis and cell motility, but not LPA breakdown. (a) Table showing cell nomenclature. (b) Western blots showing N-WASP expression in two independent KPC PDAC cell lines overexpressing GFP or GFP-N-WASP, and control (CTRL) or N-WASP CRISPR KPC PDAC cells. Red asterisk shows GFP-N-WASP. (c) Representative spider plots and Rose plots with Rayleigh test for directionality (bottom circular plots) of 35G (GFP) (i), 35N (rescue) (ii) NKPC PDAC cells, control (CTRL) (iii) and N-WASP (iv) CRISPR KPC PDAC cells in presence of 20% FBS gradient. n = 3 independent experiments, >150 cells per experiment. (d) Scatter dot plot shows the speed from KPC, two different GFP and GFP-N-WASP rescue NKPC cell lines as well as CTRL and N-WASP KPC CRISPR cells during chemotaxis assays. Mean ± SEM, n = 3, >150 cells per experiment (t-test, ****$p \leq 0.0001$). (e) LPA subspecies analysis by mass spectrometry over 60h. Graph shows an increase of the different forms of LPA in the absence of cells suggesting LPA production over time (*left panel*). When 35 NKPC PDAC cells stably transfected with GFP (plain lines) or GFP-N-WASP (dotted lines) are added the media+10% FBS, curves show a depletion of all the LPA subspecies over time indicating that all the PDAC cell lines can break down LPA (*right panel*). Mean ± SEM at each time point, (n = 3 independent experiments). (f) KPC and GFP-N-WASP rescue NKPC PDAC cells (line 35) were treated with DMSO or 10µM of KI16425 in presence of 20% FBS gradient. (i-iv) Shown here are representative spider plots and Rose plots (bottom circular plot), Rayleigh tests of these different PDAC cell lines with the different treatments (n = 4 independent experiments, >150 cells/experiment). (g) Scatter dot plot shows the speed of KPC, 35N and 15N NKPC PDAC cell lines when treated with DMSO or KI16425. Mean ± SEM, n = 4 independent experiments, >150 cells per experiment (t-test, **$p<0.01$). (h) Scatter plots show cell speed of 15N NKPC PDAC cells during random migration when treated with DMSO or KI16425. n = 3 independent experiments (t-test, not significant).

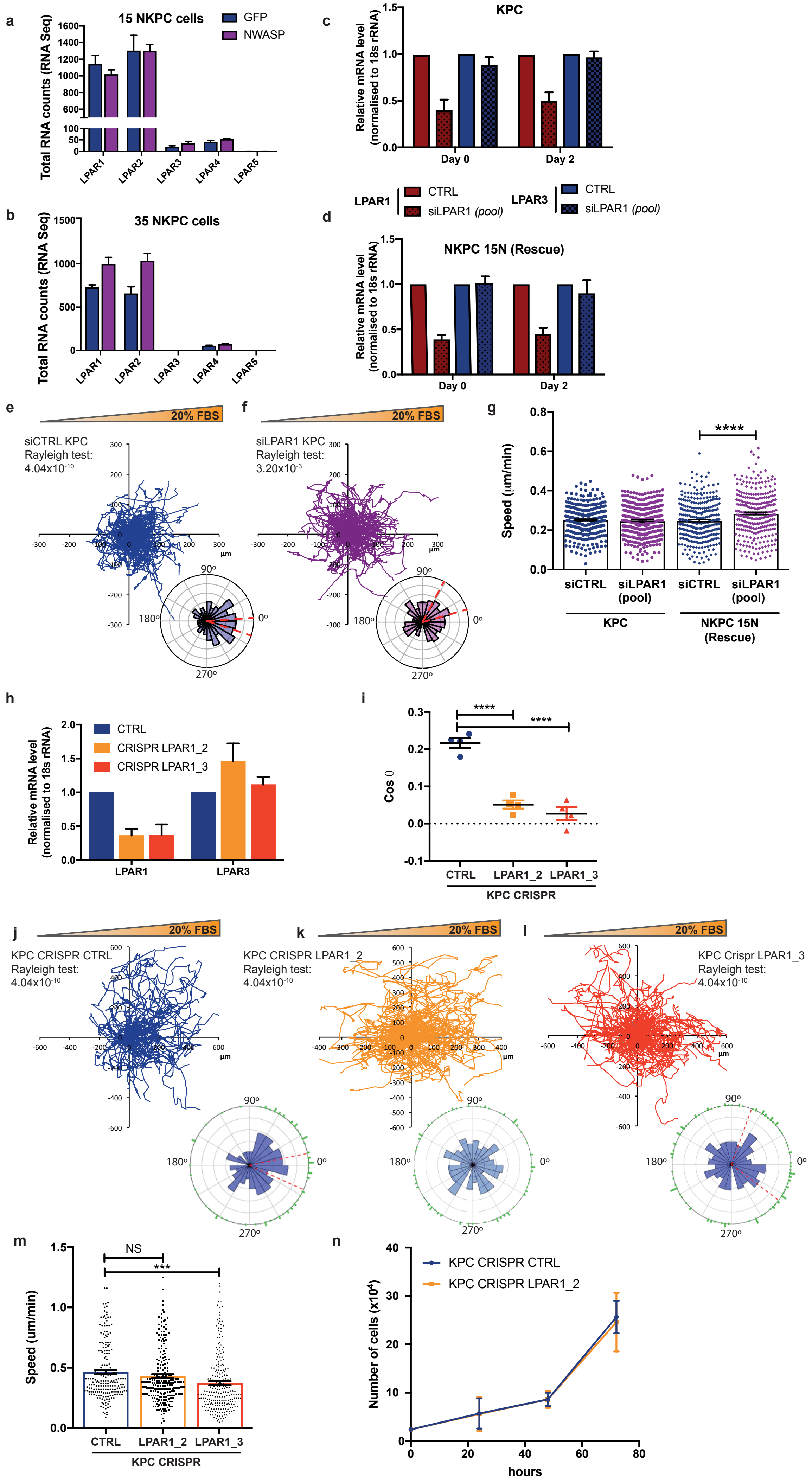

a
15 NKPC cells
GFP
NWASP
Total RNA counts (RNA Seq)
LPAR1
LPAR2
LPAR3
LPAR4
LPAR5
b
35 NKPC cells
Total RNA counts (RNA Seq)
c
KPC
Relative mRNA level (normalised to 18s rRNA)
Day 0
Day 2
LPAR1
CTRL
siLPAR1 (pool)
LPAR3
CTRL
siLPAR1 (pool)
d
NKPC 15N (Rescue)
Relative mRNA level (normalised to 18s rRNA)
Day 0
Day 2
e
20% FBS
siCTRL KPC
Rayleigh test:
4.04x10-10
f
20% FBS
siLPAR1 KPC
Rayleigh test:
3.20x10-3
g
Speed (μm/min)
siCTRL
siLPAR1 (pool)
siCTRL
siLPAR1 (pool)
KPC
NKPC 15N (Rescue)
h
Relative mRNA level (normalised to 18s rRNA)
CTRL
CRISPR LPAR1_2
CRISPR LPAR1_3
LPAR1
LPAR3
i
Cos θ
CTRL
LPAR1_2
LPAR1_3
KPC CRISPR
j
20% FBS
KPC CRISPR CTRL
Rayleigh test:
4.04x10-10
k
20% FBS
KPC CRISPR LPAR1_2
Rayleigh test:
4.04x10-10
l
20% FBS
KPC Crispr LPAR1_3
Rayleigh test:
4.04x10-10
m
NS
Speed (um/min)
CTRL
LPAR1_2
LPAR1_3
KPC CRISPR
n
KPC CRISPR CTRL
KPC CRISPR LPAR1_2
Number of cells (x10⁴)
hours

Figure S3

Figure S3 (related to Figure 2): LPAR1 is required for PDAC cell chemotaxis. (a-b) LPAR average RNA sequence reads for GFP and GFP-N-WASP rescued NKPC (line 15) (a) and line 35 (b) NKPC cell lines. Bar graphs show mean± SEM, n = 3 independent experiments. (c-d) Relative mRNA expression levels of LPAR1 and LPAR3 in KPC and 15 GFP-N-WASP NKPC cell lines transfected with CTRL or LPAR1 siRNA at the start of chemotaxis assay (day 0) or 2 days later (day 2). Bar graph shows mean ± SEM, n=4 independent experiments. (e-f) Representative Rose plots with Rayleigh test for directionality and spider plots of KPC cells transfected with siRNA control (siCTRL) (e) *vs* a pool of siRNAs targeting LPAR1 (siLPAR1 pool) (f) in presence of 20% FBS gradient. (g) Scatter dot plot shows speed of CTRL and siLPAR1 transfected KPC and 15 N-WASP rescued NKPC cells. Mean ± SEM, n = 4, >150 cells per experiment (Welch t-test, ****p<0.0001). (h) Relative mRNA expression levels of LPAR1 and LPAR3 in CTRL and 2 different guide RNA sequence targeting LPAR1: LPAR1_2 and LPAR1_3 respectively. Graph bar shows Mean ± SEM, n=4 independent experiments. (i) Cells depleted of LPAR1 lost their ability to chemotax towards the gradient as quantified by mean ± SEM (n = 4 independent experiments, >150 cells/experiment, one-way ANOVA, ****p<0.0001). (j-l) Representative rose plots with Rayleigh test for directionality and spider plots of CTRL and LPAR1 CRISPR KPC cells. (m) Scatter dot plot shows speed of CTRL and LPAR1 CRISPR KPC cells. Mean ± SEM, n = 4, >150 cells per experiment (one-way ANOVA, ***p<0.001). (n) Growth curves of CTRL and LPAR1_2 CRISPR KPC cells, n=3 independent experiments.

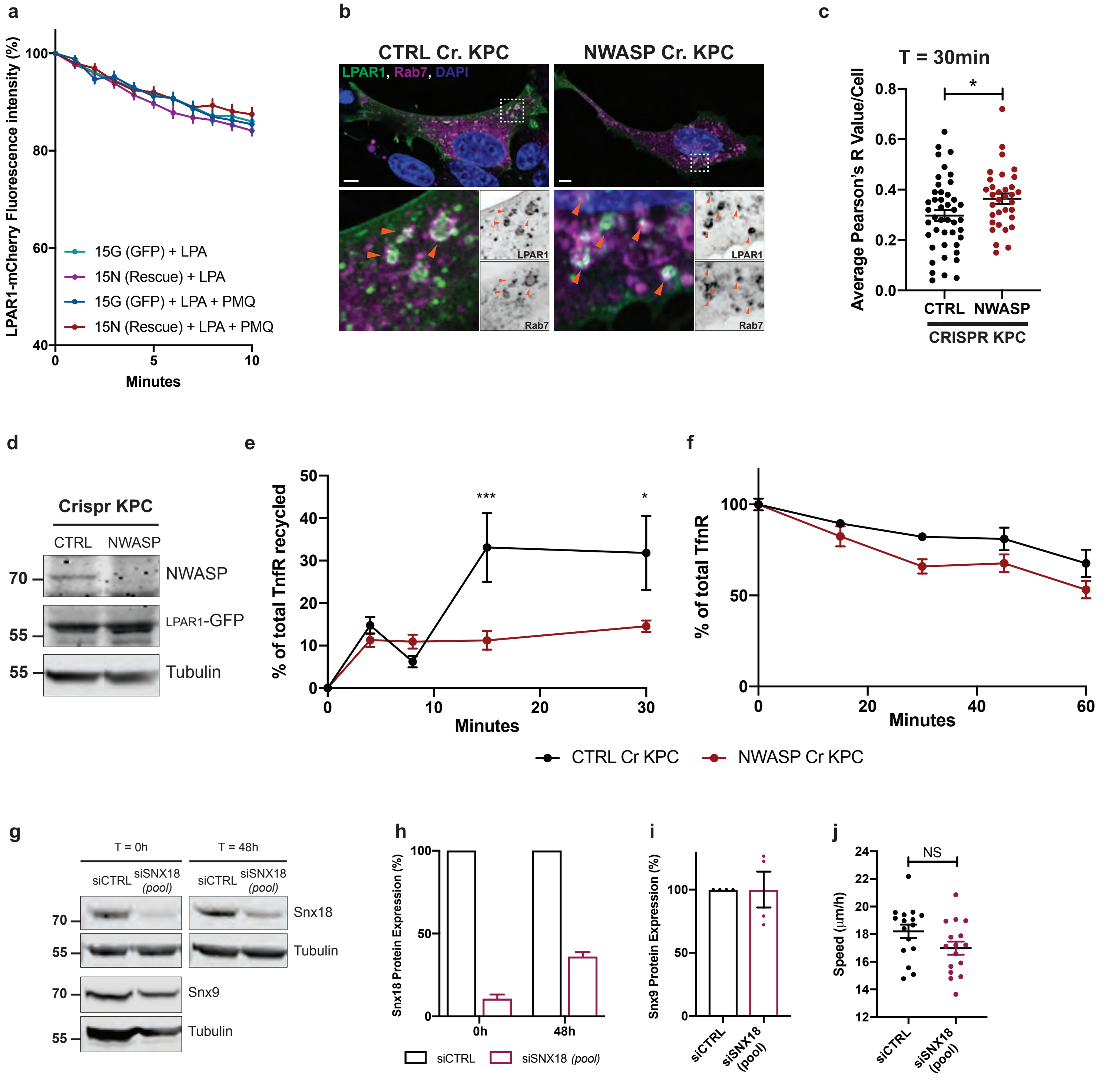

a
LPAR1-mCherry Fluorescence intensity (%)
Minutes
15G (GFP) + LPA
15N (Rescue) + LPA
15G (GFP) + LPA + PMQ
15N (Rescue) + LPA + PMQ
b
CTRL Cr. KPC
NWASP Cr. KPC
LPAR1, Rab7, DAPI
LPAR1
Rab7
c
T = 30min
Average Pearson's R Value/Cell
CTRL
NWASP
CRISPR KPC
d
Crispr KPC
CTRL
NWASP
LPAR1-GFP
Tubulin
e
% of total TnfR recycled
Minutes
f
% of total TfnR
Minutes
CTRL Cr KPC
NWASP Cr KPC
g
T = 0h
T = 48h
siCTRL
siSNX18 (pool)
Snx18
Tubulin
Snx9
h
Snx18 Protein Expression (%)
0h
48h
i
Snx9 Protein Expression (%)
j
Speed (μm/h)
NS

Figure S4

Figure S4 (related to Figure 3 and Figure 4): Loss of N-WASP affects LPAR1 recycling. (a) Quantification of LPAR1 internalisation in presence of 1μM LPA and 0.6mM primaquine (PMQ). Values are mean ± SEM, n = 3 independent experiments (>15 cells/experiment). (b) CTRL and N-WASP CRISPR KPC cells transiently transfected with LPAR1-GFP (green) and Rab7-Rfp (magenta) at 30 minutes after serum stimulation. Orange arrowheads show co-localisation between LPAR1-GFP and Rab7-RFP. DAPI, 4,6-diamino-2-phenylindole, in blue. Bottom panels show zoom of boxed images. Scale bar = 5μm. (c) Co-localisation between LPAR1-Flag and Rab7-RFP is shown as Pearson's R values after 30 minutes upon FBS stimulation. Bars show mean ± SEM, n = 3 independent experiments (>10 cells/experiment, t-test with Welch's correlation, *p≤0.05). (d) Western blot showing LPAR1-GFP expression in CTRL and N-WASP CRISPR KPC cells used for the biotinylation assays. Lysates were analysed using anti-NWASP, anti-GFP and tubulin as indicated. (e) Recycling of TfnR was determined at 4, 8, 15 and 30 minutes after serum stimulation (30 minutes endocytosis) by detection of biotinylated receptors using capture-ELISA plates coated with anti-TfnR. Values are mean ± SEM, n = 3 independent experiments (Two-way ANOVA, Sidak's multiple comparisons test, *p≤0.05, *** p≤0.001). (f) Degradation of TfnR was determined by capture-ELISA. Graphs show the percentage of the receptors remaining at 0, 15, 30, 45 and 60 minutes after serum stimulation (30 minutes endocytosis). Data are mean ± SEM, n = 3 independent experiments (Two-way ANOVA, Sidak's multiple comparisons test, **** p≤0.0001). (g-i) (g) Western blot of SNX18 in 15N (rescue) NKPC cells transfected with siCTRL or siSNX18 (pool) showing also Snx9 and tubulin. (h-i) Relative SNX18 (h) and SNX9 (i) expression were normalised to tubulin. Quantifications were done at the beginning of the experiment (0h, for SNX18 and SNX9) or 48h later (for the chemotaxis assay). Graphs show mean ± SEM, 5 independent experiments. (j) Scatter dot plot shows the speed of siCTRL and siSNX18 (pool) 15N NKPC PDAC cell. Mean ± SEM, n = 4 independent experiments, >150 cells per experiment (t-test, Not Significant).

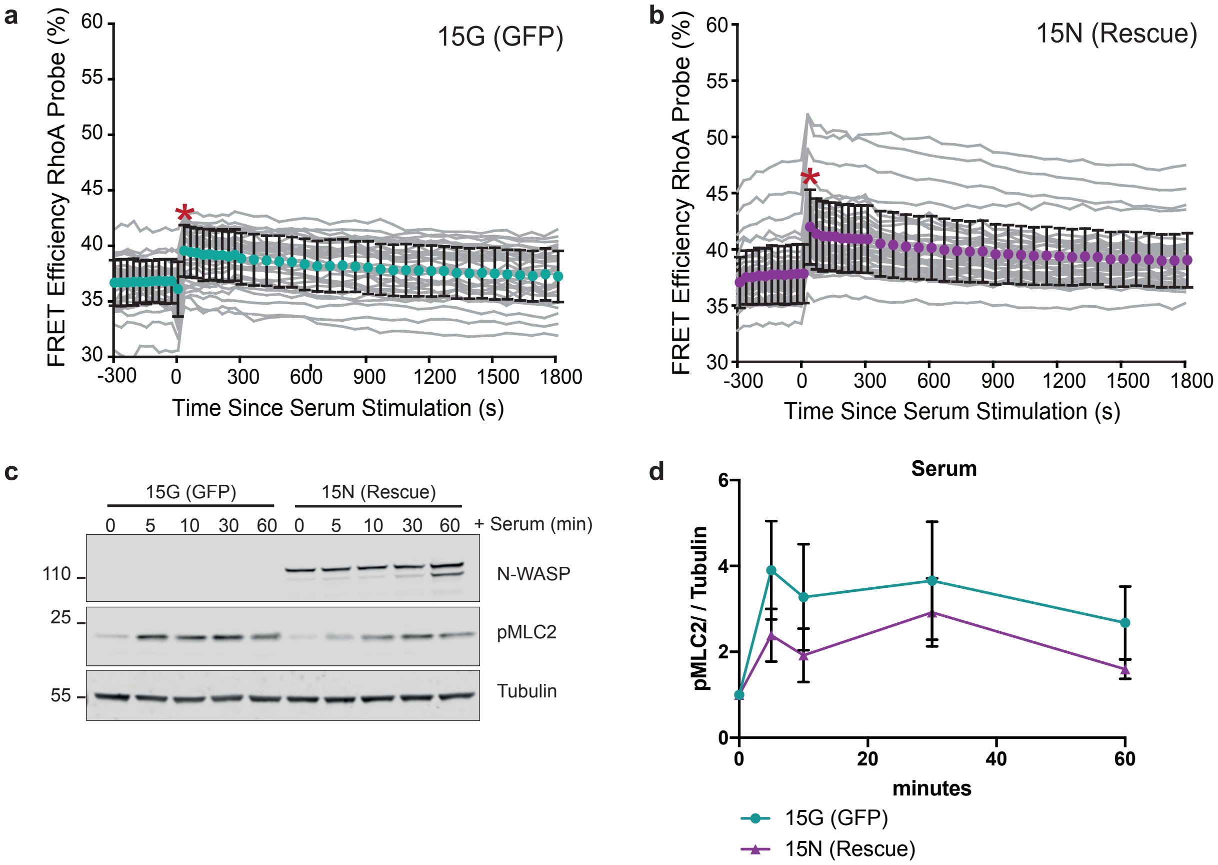
a
15G (GFP)
FRET Efficiency RhoA Probe (%)
Time Since Serum Stimulation (s)
b
15N (Rescue)
FRET Efficiency RhoA Probe (%)
Time Since Serum Stimulation (s)
c
15G (GFP)
15N (Rescue)
+ Serum (min)
N-WASP
pMLC2
Tubulin
d
Serum
pMLC2/ / Tubulin
minutes
15G (GFP)
15N (Rescue)

Figure S5

Figure S5 (related to Figure 5): Loss of N-WASP decreases RhoA activity. (a-b) Percentage of RhoA-Raichu FRET efficiency over the time after serum stimulation. Grey lines represent activity in individual cells, and the cyan and magenta circles indicate mean activity for respectively 15G (GFP-expressing) (a) and 15N (GFP-N-WASP rescue) (b) NKPC cells at each time point. Data are shown as mean ± SD (n=28 15G cells and n=40 15N cells). (c) Western blot analysis of phospho-MLC2 (Thr18/Ser19) over the time after serum stimulation in 15G (GFP) and 15N NKPC cells. Membranes were also probed with tubulin. (d) Quantification of MLC2 phosphorylation after serum stimulation in 15G (teal curve) and 15N (Rescue) (magenta curve) NKPC cells over the time. Relative (Thr18/Ser19) MLC2 phosphorylation was normalised to tubulin. Graphs show mean ± SEM, 5 independent experiments.

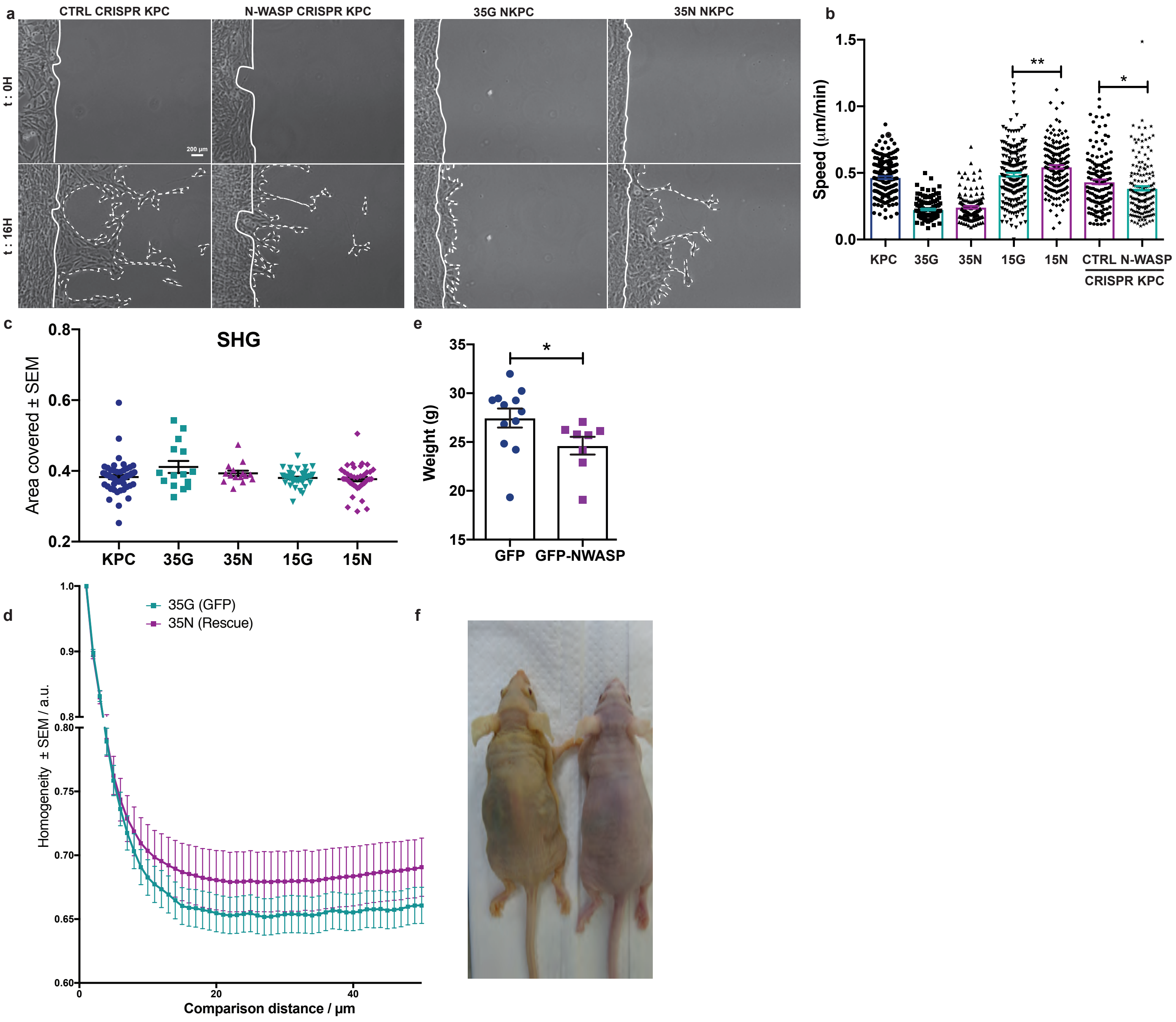
a
CTRL CRISPR KPC
N-WASP CRISPR KPC
35G NKPC
35N NKPC
t : 0H
t : 16H
200 µm
b
Speed (µm/min)
1.5
1.0
0.5
0.0
**
*
KPC
35G
35N
15G
15N
CTRL
N-WASP
CRISPR KPC
c
SHG
Area covered ± SEM
0.8
0.6
0.4
0.2
KPC
35G
35N
15G
15N
e
Weight (g)
35
30
25
20
15
*
GFP
GFP-NWASP
d
35G (GFP)
35N (Rescue)
Homogeneity ± SEM / a.u.
1.0
0.9
0.8
0.80
0.75
0.70
0.65
0.60
0
20
40
Comparison distance / µm
f

Figure S6

0º

Figure S6 (related to Figure 6): Loss of N-WASP impairs invasion and matrix remodelling through LPA signaling. (a) Representative images of control (CTRL) or N-WASP CRISPR KPC PDAC cells and 35 NKPC PDAC cells stably transfected with GFP (35G) or GFP-N-WASP (35N) at t=0h or 16h after invading Matrigel. Plain and dotted white lines show limits between Matrigel and invading cells at t=0h and t=16h respectively. Scale bar = 200µm. See also Supplementary Movie S8. (b) Scatter plot shows speed of the cells during Matrigel invasion. Mean ± SEM, n = 4 independent experiments with >30 cells quantified each time (Mann-Whitney test or Welch's t-test were used to compare 35G *vs* 35N NKPC PDAC cells and CTRL *vs* N-WASP CRISPR KPC PDAC cells, and 15G *vs* 15N NKPC PDAC cells respectively: *p≤0.05, **p≤0.01). (c) Total SHG signal per area. Mean ± SEM. (Mann-Whitney test or Welch's t-test were used to compare 35G *vs* 35N NKPC PDAC cells and 15G *vs* 15N NKPC PDAC cells respectively, not significant). (d) Gray-level correlation matrix texture analysis of Angular Second Moment (n=3 independent experiments, each n=10 fields of view). Mean of pixels ± SEM at each distance. Scale Bar = 50µm. (e) Weight of mice 15 days after injection. Mean ± SEM (Mann-Whitney U test, *p<0.05). (f) Representative image of mice injected with 15N (rescue, *left*) or 15G (GFP, *right*) NKPC PDAC cells. Note that mouse injected with 15N NKPC PDAC cells presents a yellowish tinge of the skin, known as jaundice, caused by an obstruction of the bile duct.
